# Crystal structure-based discovery of a novel synthesized PARP1 inhibitor (OL-1) with apoptosis-inducing mechanisms in triple-negative breast cancer

**DOI:** 10.1038/s41598-016-0007-2

**Published:** 2016-12-05

**Authors:** Leilei Fu, Shuya Wang, Xuan Wang, Peiqi Wang, Yaxin Zheng, Dahong Yao, Mingrui Guo, Lan Zhang, Liang Ouyang

**Affiliations:** 1 State Key Laboratory of Biotherapy and Cancer Center, West China Hospital, Sichuan University, and Collaborative Innovation Center of Biotherapy, Chengdu, 610041 China; 2 Northwestern University, Feinberg School of Medicine, 303 East Chicago Avenue, Chicago, Illinois 60611 USA; 3 State Key Laboratory of Oral Diseases, West China Hospital of Stomatology, Sichuan University, Chengdu, 610041 China

## Abstract

Poly (ADP-ribose) polymerase-1 (PARP1) is a highly conserved enzyme focused on the self-repair of cellular DNA damage. Until now, numbers of PARP inhibitors have been reported and used for breast cancer therapy in recent years, especially in TNBC. However, developing a new type PARP inhibitor with distinctive skeleton is alternatively promising strategy for TNBC therapy. In this study, based on co-crystallization studies and pharmacophore-docking-based virtual screening, we discovered a series of dihydrodibenzo[b,e]-oxepin compounds as PARP1 inhibitors. Lead optimization result in the identification of compound OL-1 (2-(11-(3-(dimethylamino)propylidene)-6,11- dihydrodibenzo[b,e]oxepin )-2-yl)acetohydrazide), which has a novel chemical scaffold and unique binding interaction with PARP1 protein. OL-1 demonstrated excellent potency (inhibiting PARP1 enzyme activity with IC_50_ = 0.079 μM), as well as inhibiting PARP-modulated PARylation and cell proliferation in MDA-MB-436 cells (*BRAC1* mutation). In addition, OL-1 also inhibited cell migration that closely related to cancer metastasis and displayed remarkable anti-tumor efficacy in MDA-MB-436 xenograft model without apparent toxicities. These findings highlight a new small-molecule PAPR1 inhibitor (OL-1) that has the potential to impact future TNBC therapy.

## Introduction

Poly (ADP-ribose) polymerase-1 (PARP1) is a highly conserved enzyme focused on the self-repair of cellular DNA damage, participating in several biological processes including apoptosis, chromosome stability, gene amplification, transcriptional regulation and cell division^1, 2^. When DNA damage occurs, PARP1 senses and binds to the site of Single-strand breaks (SSBs) and becomes catalytically activated. It utilizes nicotinamide adenine dinucleotide (NAD^+^) as substrate to form branching chains of poly (ADP-ribose) (PAR) onto PARP1 itself as well as other nuclear proteins or enzymes including histones, DNA topoisomerases, ligases and polymerases^3, 4^. Synthesized PAR chains recruit X-ray repair cross-complementing protein 1 (XRCC1), DNA ligase III and DNA polymerase β to DNA damage sites, subsequently mediating base excision repair (BER)^5^. Inhibition of PARP1 will lead to the accumulation of SSBs and stalling of DNA repair machinery, finally resulting in double-strand breaks (DSBs)^6^. Interestingly, over-expressed PARP1 has been demonstrated in various cancers such as melanomas, glioblastoma and breast cancer^7–11^. Moreover, high expression level of PARP1 was found closely related with triple-negative breast cancer (TNBC)^12^. Consequently, targeting PARP1 and inhibiting its relevant biological function may be another avenue of breast cancer therapy, especially for TNBC.

**Figure 1:**
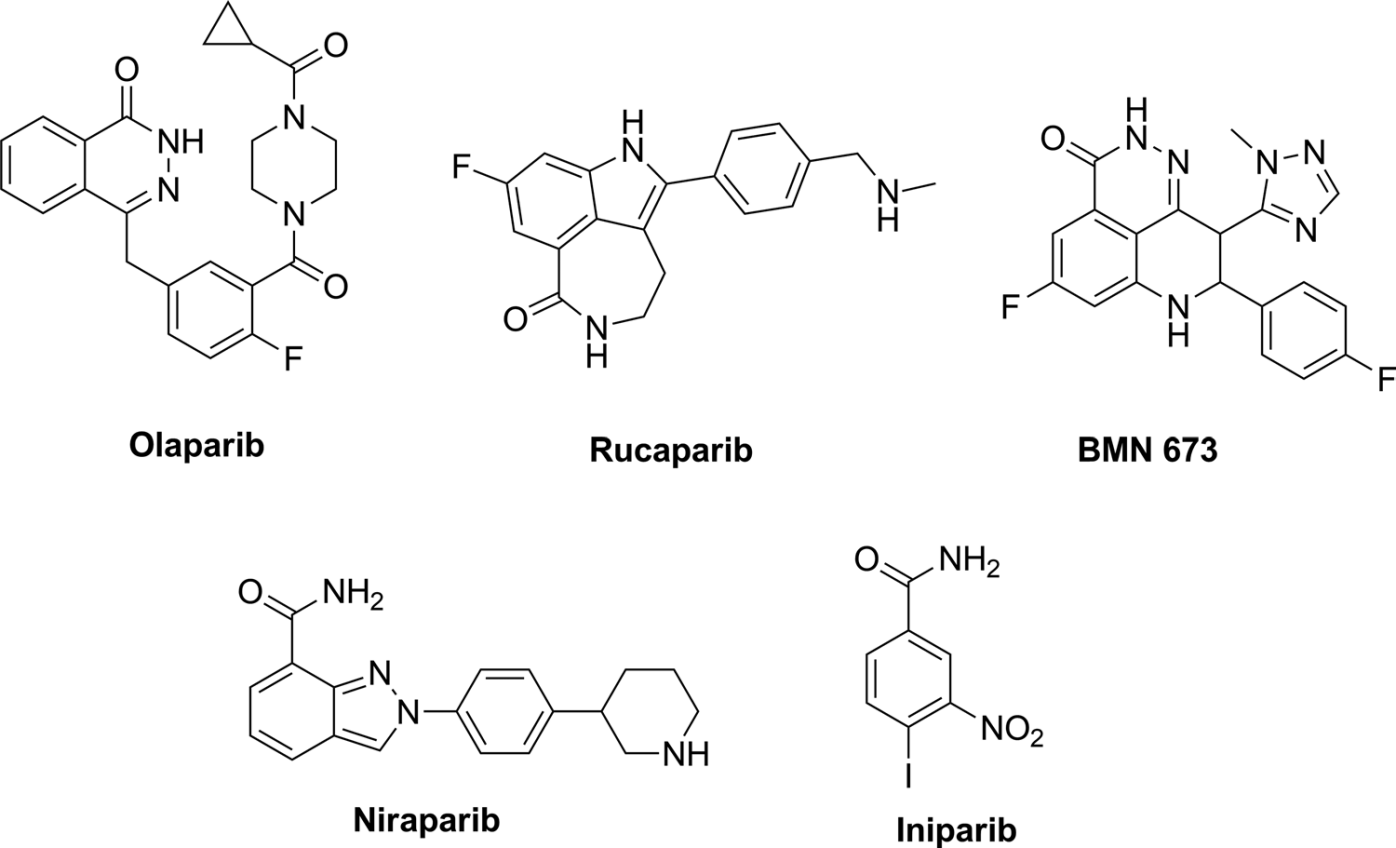
PARP inhibitors in clinical trial.

Previous studies have been reported that inhibition of PARP1 leads to synthetic lethality in some BRCA1/2 mutant cancers (including ovarian and breast cancer), which could be specifically targeted by PARP1 inhibitors^13^. Currently, various PARP inhibitors, such as Olaparib, Rucaparib, BMN-673, Niraparib and Iniparib (Fig. 1), are under development indifferent stages of clinical trial^14–20^. From a chemical point of view, most chemical scaffolds of PARP inhibitors contain amide structure, more new chemical structures can be found in the future^21, 22^; From a biological point of view, although these PARP inhibitors have high PARP1/2 inhibition and anti-tumor activity; however, long-term drug administration will accompany with drug resistance, leading to tumor recurrence and metastasis^23^. Thus, in addition to explore the in-depth drug resistance mechanism of existing inhibitors, as well as the relationship between PARP-mediated signaling pathways and tumor specificity, developing a new type PARP inhibitor with improved therapeutic efficacy and lower toxicity is alternatively promising strategy for TNBC therapy.

With the rapid development of computational methods and structural biology, many studies successfully identifying epigenetic inhibitors using pharmacophore-docking-based virtual screening and co-crystallization studies have been reported^24–26^. In this study, by constructing a pharmacophore of PARP1 inhibitor and screening a new chemical skeleton through co-crystallization studies, we designed and synthesized several series of PARP1 inhibitors, then identified a novel PARP1 inhibitor (OL-1). This inhibitor could significantly induce cell death and inhibit cell migration in *BRAC1* mutant MDA-MB-436 cells with potent anti-tumor efficacy *in vivo*. These findings highlight a new small-molecule PAPR1 inhibitor (OL-1) that has the potential to impact future TNBC therapy.

## Results and Discussion

### Co-crystallization screening and structure-based pharmacophore of PARP1/inhibitor complex

**Figure 2:**
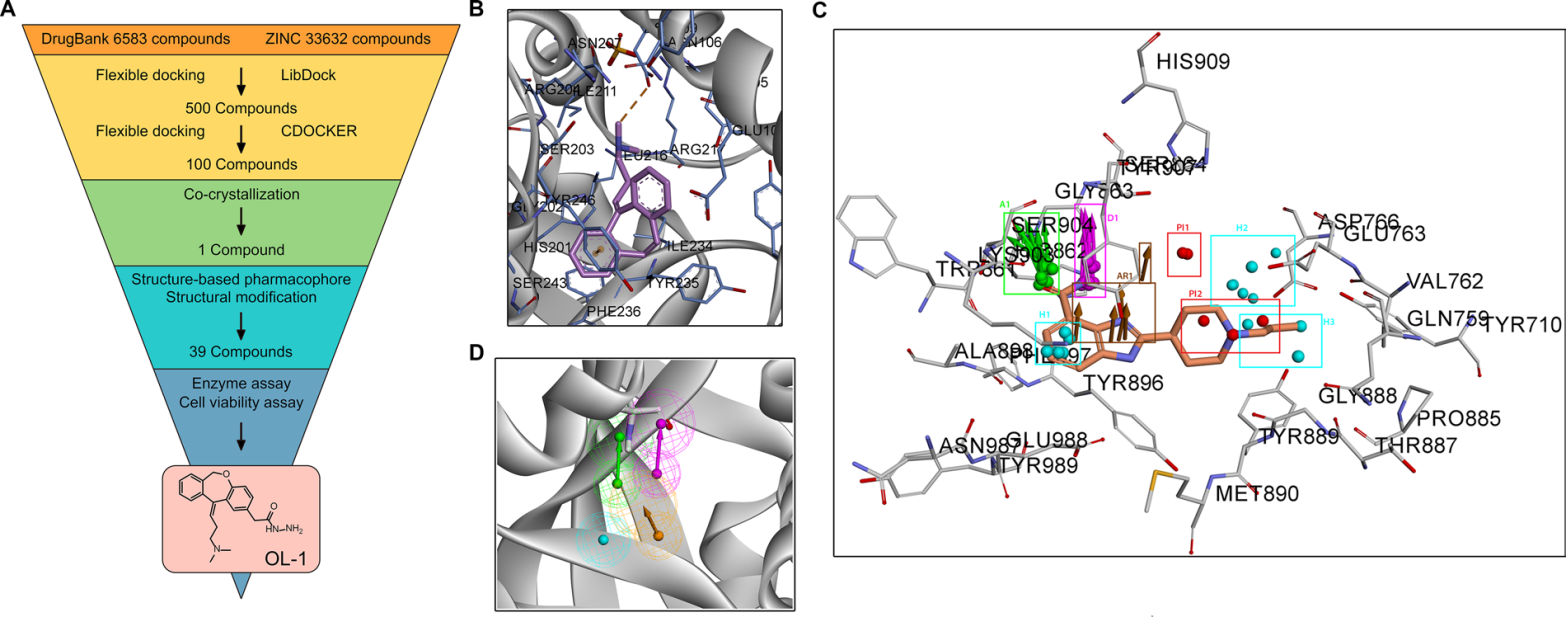
Crystal structure, pharmacophore models construction and molecular docking of PARP1 inhibitors. (**A**) Virtual screening schematic model for the discovery process of novel PARP1 inhibitors. (**B**) Candidate PARP1 inhibitor PA-10 bind to the NAD^+^ binding site. (**C**) Comprehensive structure-based pharmacophore features of PARP inhibitors. (**D**) The four common structure-based pharmacophore model.

Numbers of PARP inhibitors have been reported over the past several years, such as Olaparib, Rucaparib, BMN 673, Niraparib and Iniparib (Fig. 1)^27–29^. These previous work had well described that PARP inhibitors occupy the nicotinamide pocket in the NAD^+^ binding site of PARP1, forming key hydrogen bonds and π-π interactions. Firstly, we used virtual screening of chemical libraries that based upon Drugbank and ZINC databases, searching for novel leading compounds with distinctive skeleton (Fig. 2A). Top500 hits were selected by LibDock protocol in the first step. Subsequently, Top100 (PA-1 ~ PA-100) hits were further determined by CDOCKER protocol and selected for co-crystallization screening. As a result, only one compound from Drugbank database (DB00321) named as PA-10 (3-(10,11-dihydro-5H-dibenzo[a,d][7]annulen-5-ylidene)-N,N-dimethylpropan-1-amine) bound to the nicotinamide pocket of PARP1 (PDB ID code 5HA9) in the co-crystallization screening (Fig. 2B). To explore how to modify the leading compound, we constructed the structure-based pharmacophore including ten reported co-crystal structures of PARP inhibitors. The detected pharmacophore features were shown in Table 1 and Fig. 2C. Among these features, four of them were found as common in these complexes, including A1 (hydrogen bond acceptor), D1 (hydrogen bond donor), AR1 (ring aromatic) and H1 (hydrophobic) (Fig. 2D). Therefore, we can further modify the leading compound by increasing the length of carbon chain; increasing substituent containing hydrogen bond donor and changing of aromatic skeleton. And all newly synthesized compounds were designed according to abovementioned structure-based pharmacophore features.

**Table 1:** Analyses of pharmacophore features based on ten co-crystal structures of PARP1 inhibitors obtained from the Protein Data Bank (PDB).

PDB No.	1UK0	1UK1	1WOK	2RCW	3L3L	3L3M	4HHY	4L6S	4ZZZ	5A00
resolution	3.0 Å	3.0 Å	3.0 Å	2.8 Å	2.5 Å	2.5 Å	2.36 Å	2.2 Å	1.9 Å	2.75 A
ligand	FRM	FRQ	CNQ	AAI	L3L	A92	15R	1WQ	FSU	D7N
Release date	2004/1/27	2004/1/27	2005/3/15	2008/9/23	2010/12/22	2010/6/23	2013/3/27	2013/8/7	2015/8/12	2015/8/12
pharmacophore model features										
A1	√	√	√	√	√	√		√	√	√
D1	√	√	√	√	√	√	√	√	√	√
AR1			√	√	√	√		√	√	
H1	√	√	√		√	√	√		√	√
H2	√	√					√			
H3			√	√						√
PI1	√	√						√		
PI2				√		√				

### Chemistry

**Figure 3:**
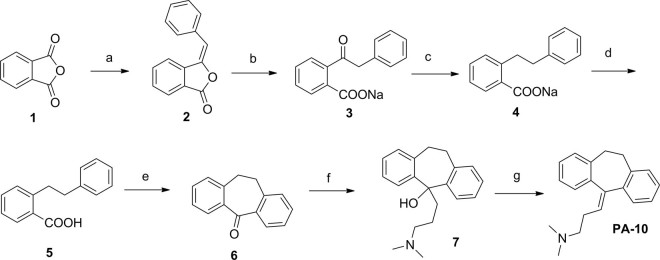
General Synthesis of compound PA-10. Reagents and conditions: (**a**) Phenylacetic acid, CH_3_COONa, fusion at 200 °C, 5 h; (**b**) MeOH, NaOH; (**c**) PEG, hydrazine hydrate, NaOH, 110 to 180 °C; (**d**) HCl; (**e**) PPA, 100 °C; (**f**) THF, (3-(dimethylamino)propyl)magnesium chloride; (**g**) EtOH, con.HCl, reflux.

As outlined in Fig. 3, 3-(10,11-dihydro-5H-dibenzo[a,d][7]annulen-5-ylidene)-N,N-dimethylpropan-1-amine (PA-10), prepared from commercially available isobenzofuran-1,3-dione (**1**) which was used as the key intermediate by following a literature procedure: Phthalic anhydride and phenylacetic acid were reacted in present of sodium acetate, then the product of Friedel-Crafts reaction was hydrolyzed under alkaline conditions. A Wolff-Kishner-Huang reduction was carried on and the intermediate was cyclizing in acidic conditions. The final product was obtained by Grignard reaction after hydrolysis and dehydration.

**Figure 4:**
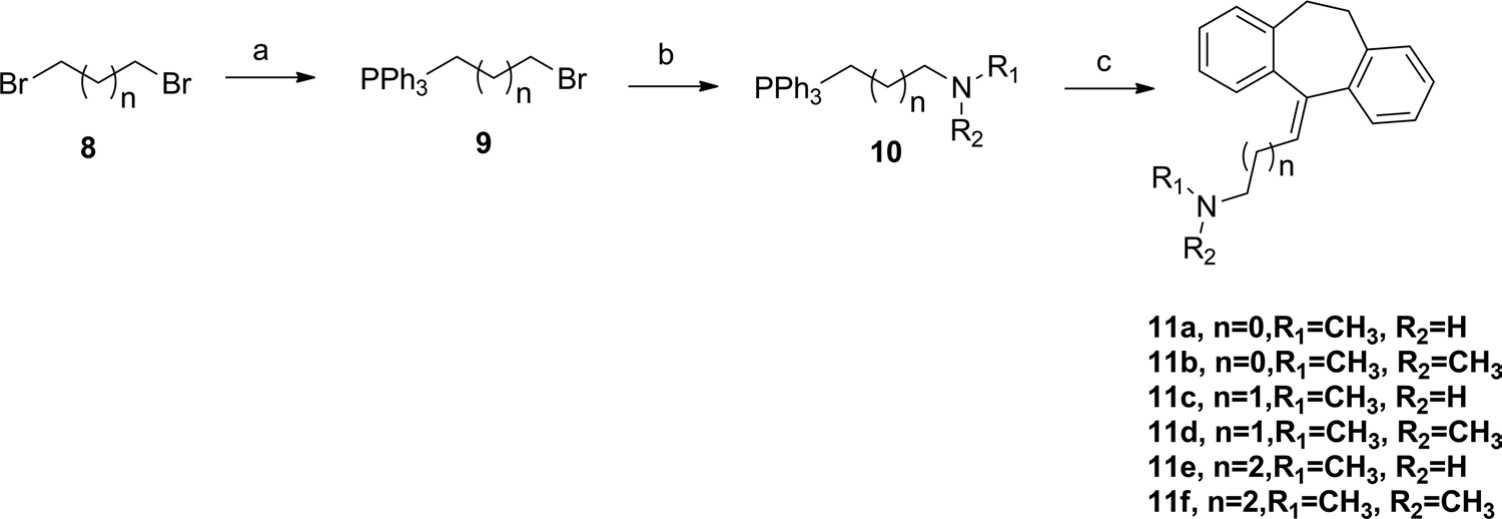
Synthesis of 10,11-dihydro-5H-dibenzo[a,d][7]annulen-5-ylidenederivatives. Reagents and conditions: (**a**) toluene, Ph_3_P, reflux; (**b**) EtOH, amines, 70 °C; (**c**) THF, **6**, n-BuLi, −10 °C to reflux.

**Figure 5:**
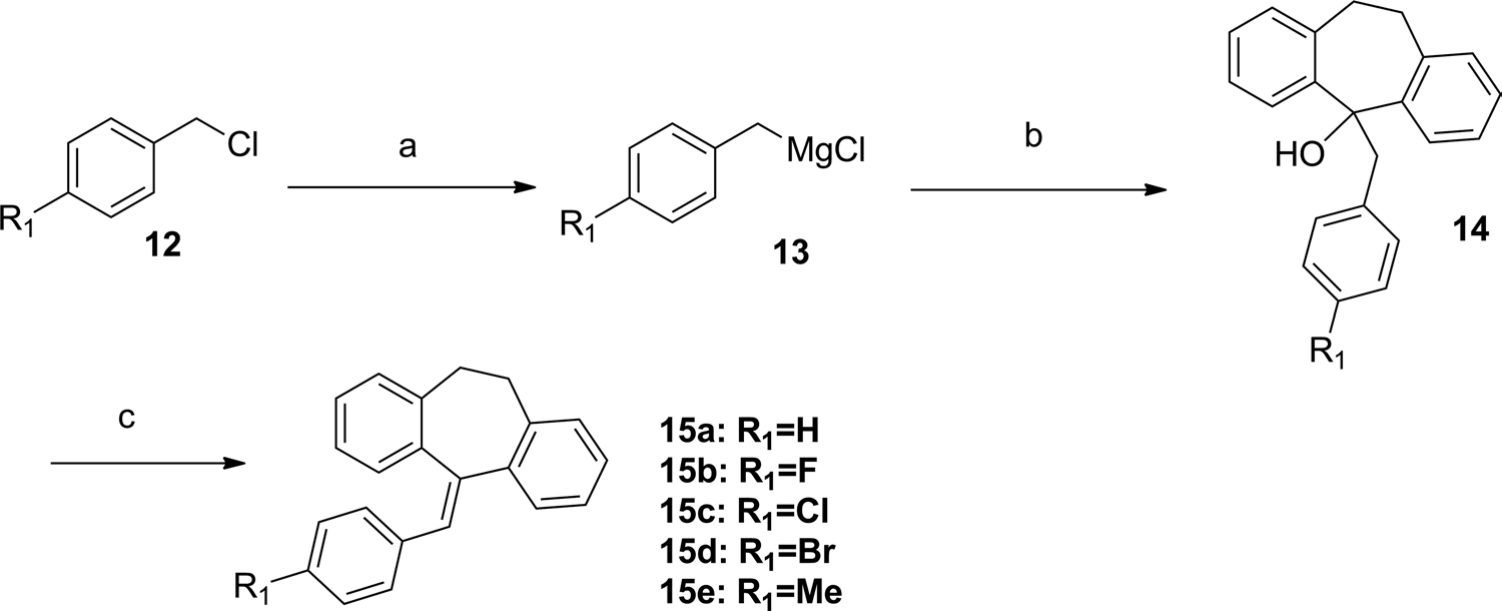
Synthesis of 10,11-dihydro-5H-dibenzo[a,d][7]annulenederivatives. Reagents and conditions: (**a**) THF, Mg, I_2_; (**b**) THF, **6**; (**c**) HCl.

Meanwhile, condensation of 1,2-dibromoethane (or propane, butane) with Ph_3_P in toluene provided compound **9**, subsequent substituted by different amines in 74–85% overall yield respectively. Then, a witting reaction occurred in present of compound **10** with n-BuLi in −10 °C provided 10,11-dihydro-5H-dibenzo[a,d][7]annulen-5-ylidene derivatives (**11a–f**)in relatively high yields 82–91% (Fig. 4). 5-benzylidene-10,11-dihydro-5H-dibenzo[a,d][7]annulene derivatives (**15a–e**) were prepared by (chloromethyl)benzene derivatives (**12**) reacting with Mg and I_2_, then condensation of the Grignard reagents (**13**) with compound **10** (Fig. 5). Inserting varies R1 moieties to the connecting linkage yielded compounds **15a–e** in 45–78% overall yield.

**Figure 6:**
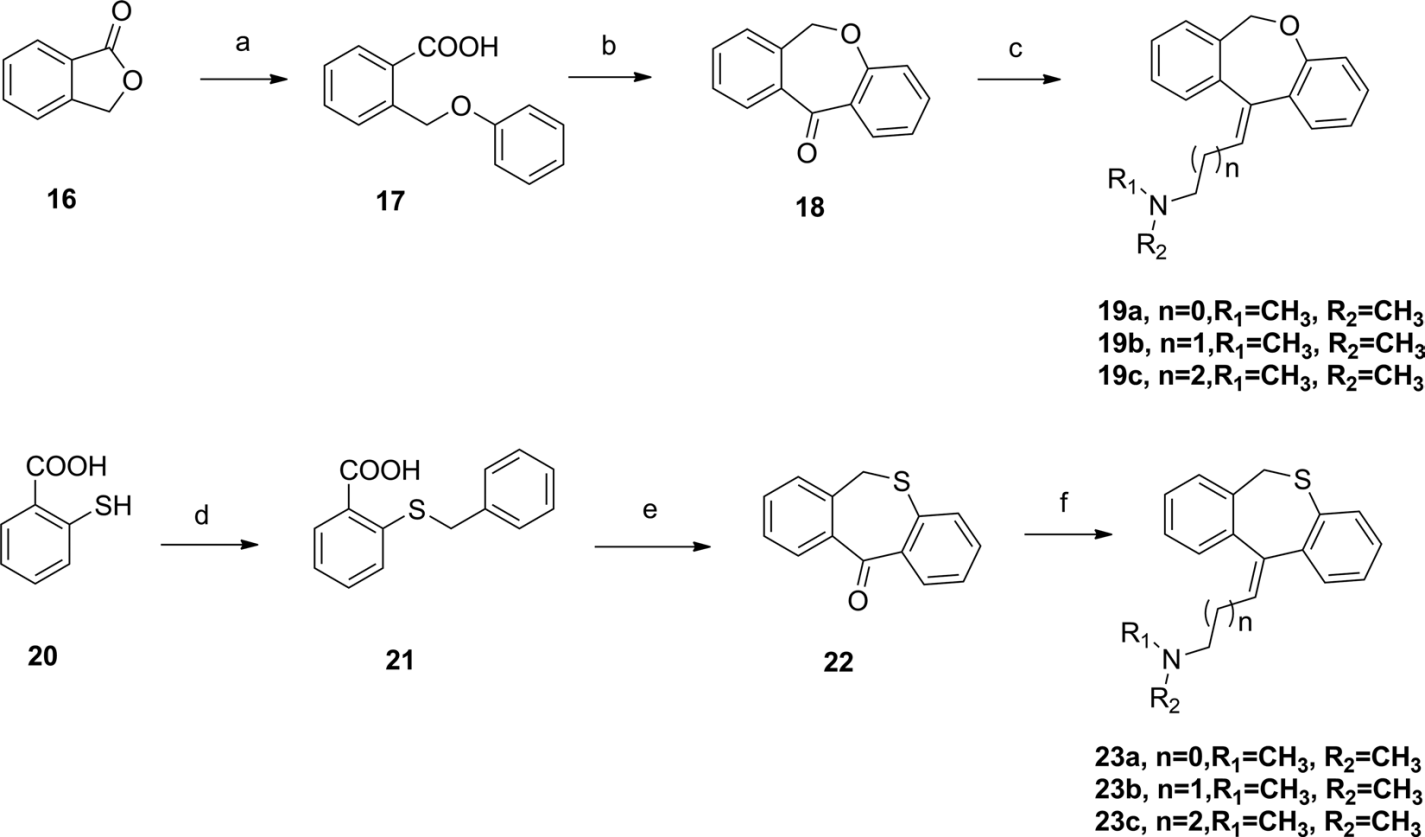
Synthesis of dibenzo[b,e]oxepin-11(6H)-ylidene and dibenzo[b,e]thiepin-11(6H)-ylidenederivatives. Reagents and conditions: (**a**) KOH, Xylene, reflux, then HCl, H_2_O; (**b**) CH_2_Cl_2_, trifluoroacetic anhydride, BF_3_.Et_2_O,40 °C; (**c**) THF, **10**, n-BuLi; (**d**) EtOH, NaOH, overnight; (**e**) PPA, 100 °C; (**f**) THF, **10**, n-BuLi.

To explore the impact of structure modifications in mother structures, as well as the framework reconstruction, compound **19** and **23** derived from dibenzo[b,e]oxepin-11(6H)-one (**18**) and dibenzo[b,e]thiepin-11(6H)-one (**22**) was also prepared (Fig. 6). Isobenzofuran-1(3H)-one (**16**) was treated with KOH in the temperature of the xylene reflux, then acidified with HCl to obtain compound **17** in 41% yield. The intermediate was then cyclizing in present of trifluoroacetic anhydride and BF_3_.Et_2_O to obtain compound **18** in 90% yield. A witting reaction similar to previous descriptions occurred in present of compound **10** with n-BuLi in −10 °C provided dibenzo[b,e]oxepin-11(6H)-ylidene derivatives (**19a–c**) in relatively high yields. Compound **22** was synthesized by a similar cyclization reaction in present of PPA and the final products dibenzo[b,e]thiepin-11(6H)-ylidene derivatives (**23a–c**) were obtained by a witting reaction similar to previous descriptions with n-BuLi in −10 °C.

**Figure 7:**
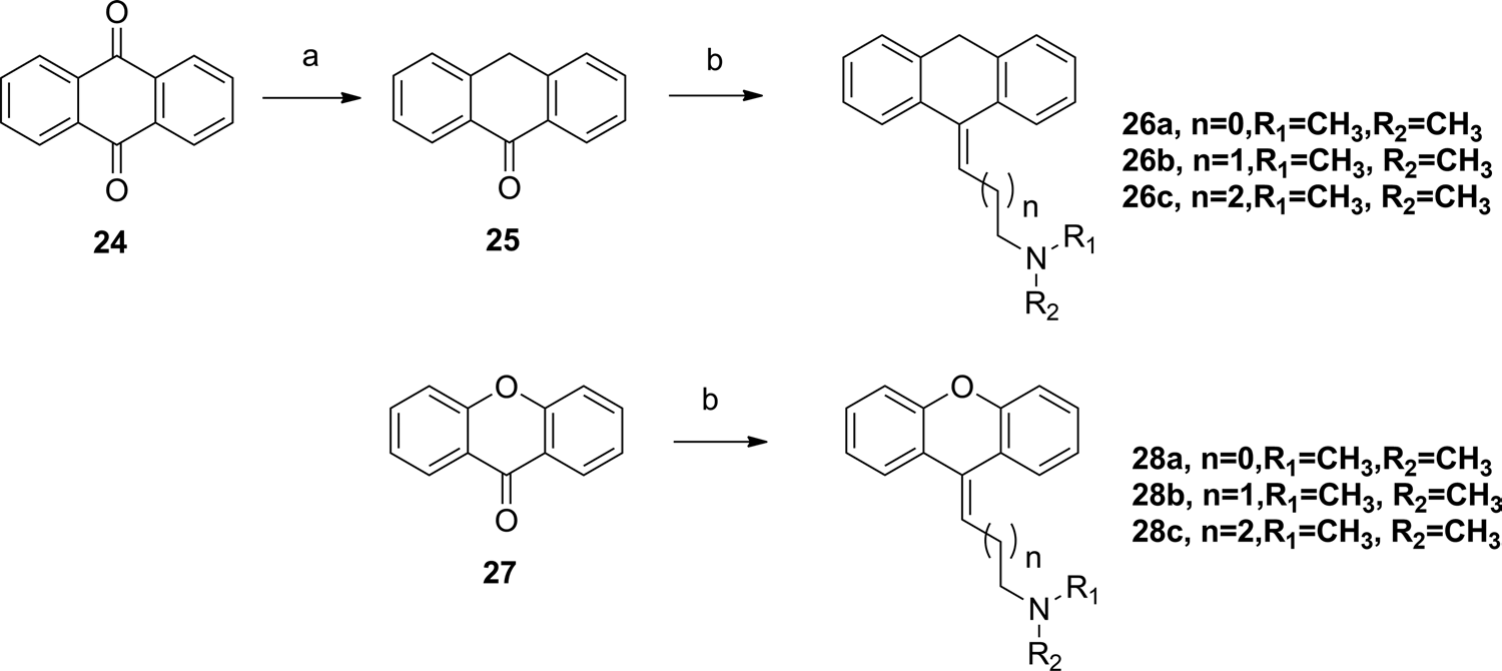
Synthesis of anthracen-9(10H)-ylidene and 9H-xanthen-9-ylidenederivatives. Reagents and conditions: (**a**) SnCl_2_, HCl, AcOH, reflux; (**b**) THF, **10**, n-BuLi.

At the same time, anthracen-9(10H)-ylidene derivatives (**26a–c**), 9H-xanthen-9-ylidene (**28a–c**), devoid of one carbon atom in the mother structures, were prepared from commercially available and respective ketones in three steps similar to the above synthetic description (Fig. 7).

**Figure 8:**
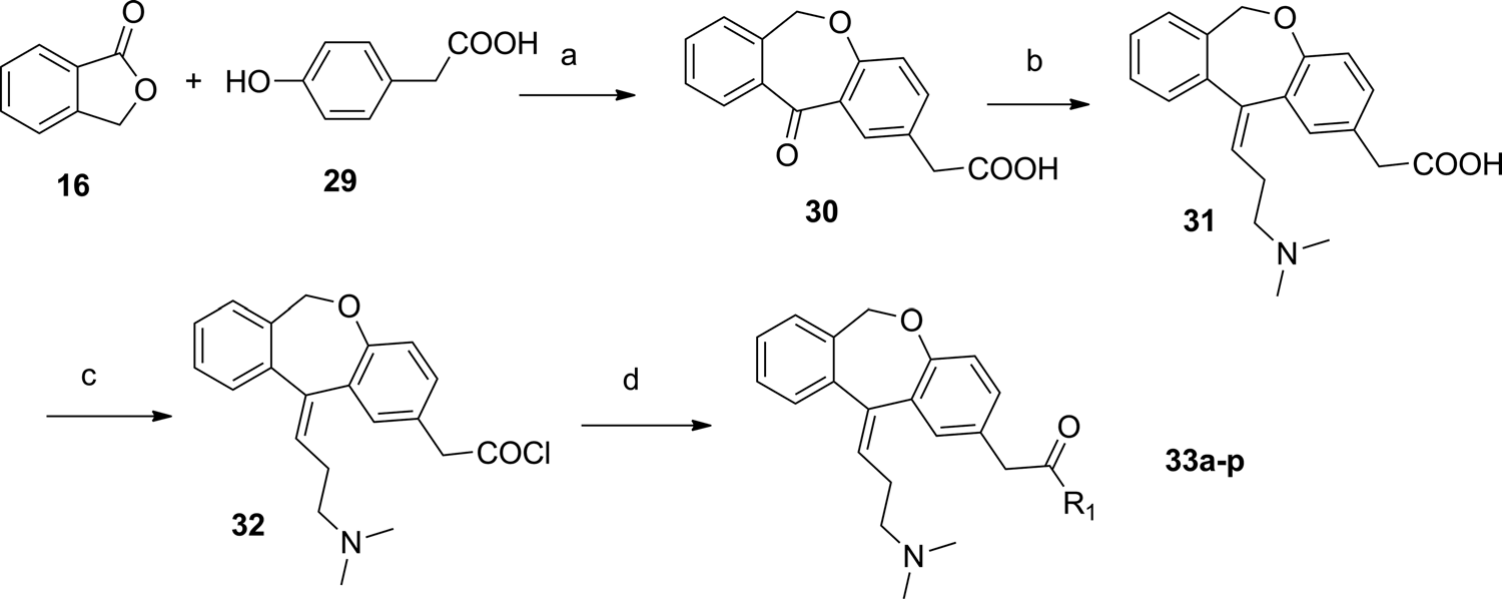
Synthesis of 3-(dimethylamino)propylidene)-6,11-dihydrodibenzo[b,e]oxepinderivatives. Reagents and conditions: (**a**) DMF, MeONa, 120 °C; then, PPA, AcOH, 75 °C; (**b**) THF, **10**, n-BuLi; (**c**) CH_2_Cl_2_, SOCl_2_, r.t.; (**d**) CH_2_Cl_2_, amine.

Figure 8 showed the synthetic route of 2-substituted 3-(dimethylamino)propylidene)-6,11-dihydrodibenzo[b,e]oxepin derivatives (**33a–p**): compound **16** was reacted with 2-(4-hydroxyphenyl)acetic acid (**29**) in present of MeONa in DMF, then acidified with HCl. The intermediate was then cyclizing in present of PPA in AcOH to obtain compound **30** in 85% yield.

### Structural modification and structure activity relationship analysis

**Figure 9:**
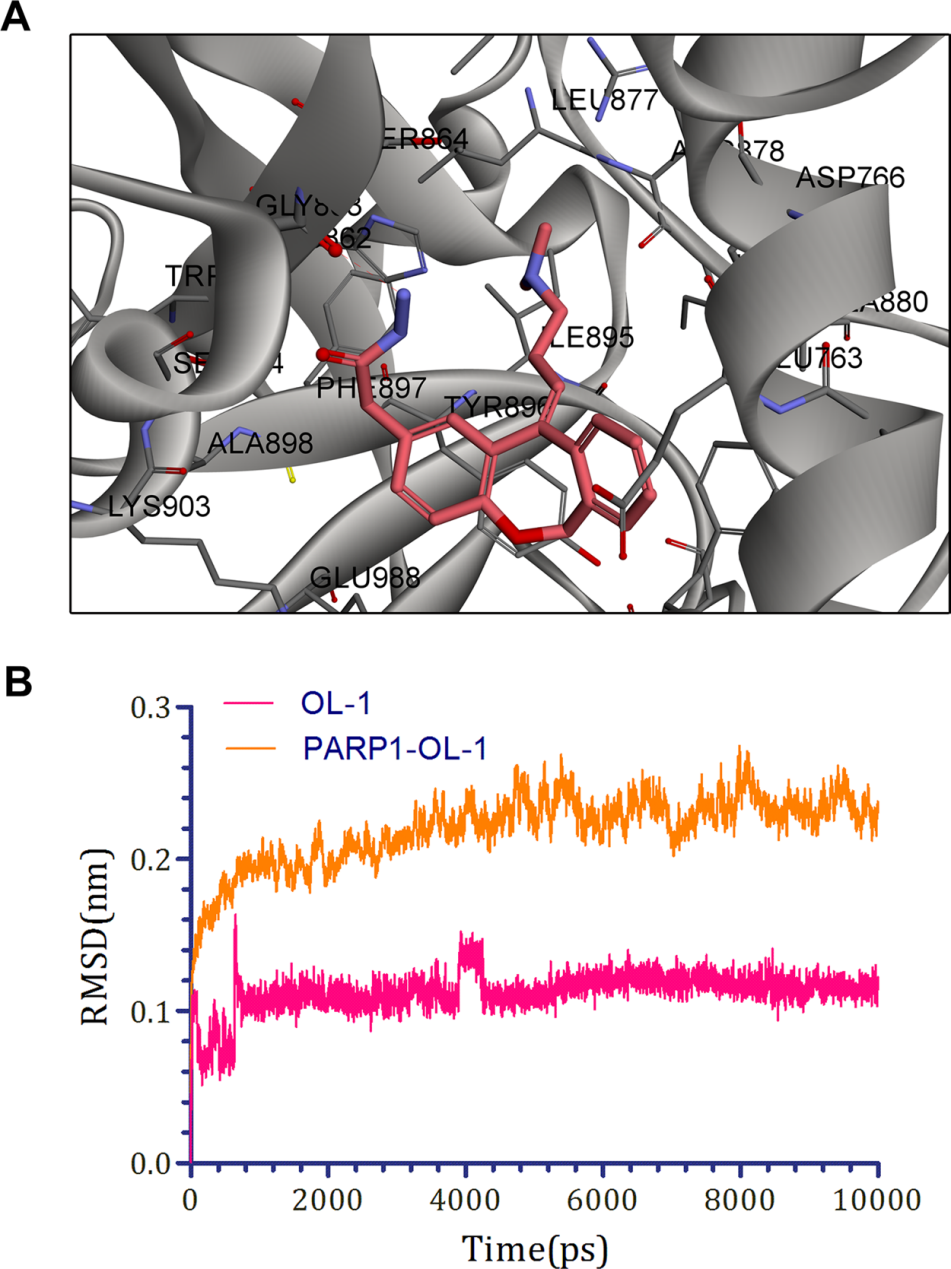
Molecular docking and molecular dynamics (MD) simulations of candidate PARP1 inhibitor OL-1. (**A**) Molecular docking of PARP1/OL-1 complex indicated that two hydrogen bonds were formed with GLY863. (**B**) Molecular dynamics (MD) simulations of OL-1 binding to PARP1. The binding conformation was stabilized after 10 ns simulation.

**Figure 10:**
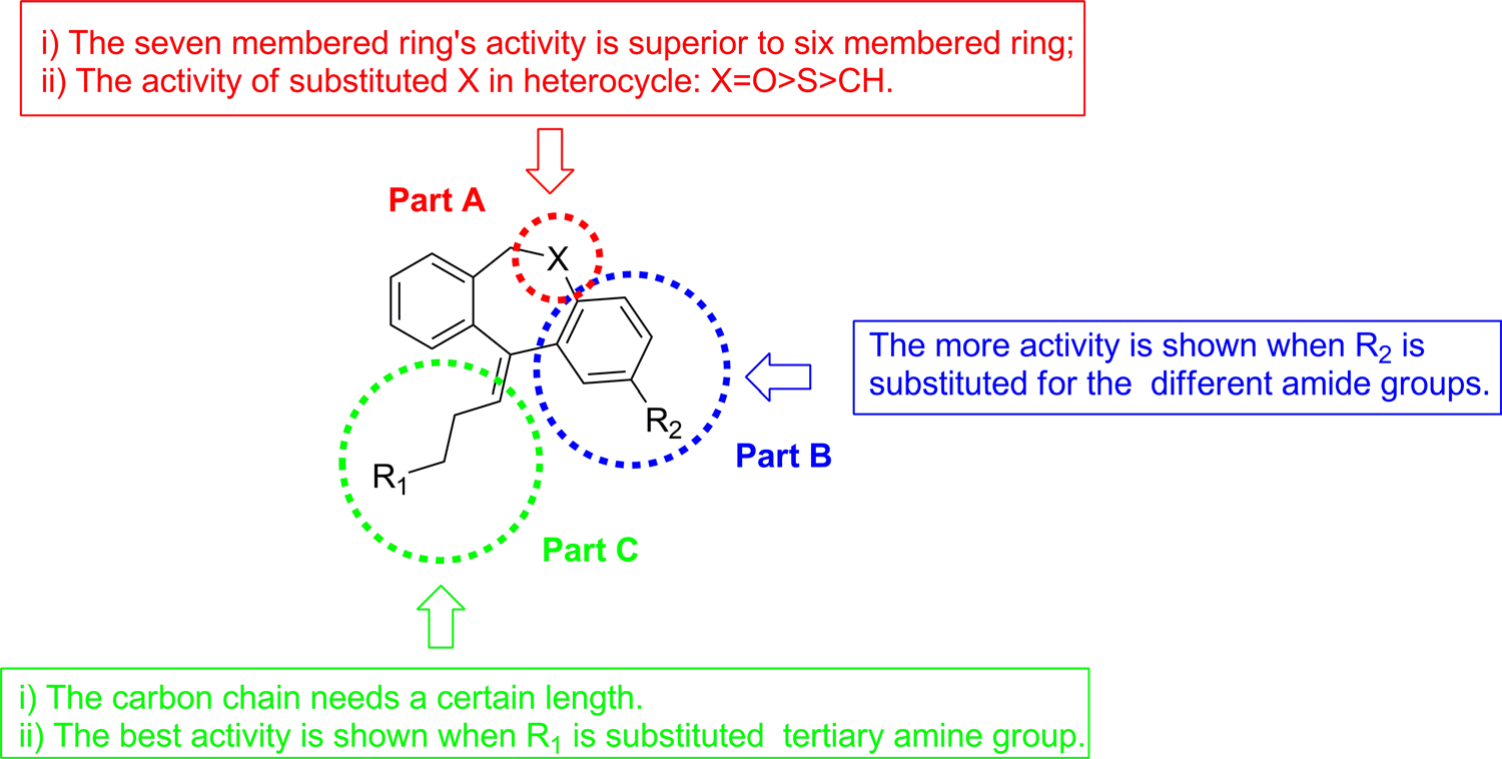
Regions subjected to separate SAR investigation.

All synthesized compounds were tested to determine their PARP1 inhibition activities, and all compounds were further evaluated by cell viability assay in MDA-MB-436 cells (*BRAC1* mutant breast cancer). The clinical small molecular PARP1 inhibitors Iniparib and Olaparib were used as the reference compound. First, 10,11-dihydro-5H-dibenzo[a,d][7]annulen-5-ylidene derivatives (**11a–f**) with a N,N-disubstited amino group attached 10,11-dihydro-5H-dibenzo[a,d][7]annulen-5-ylidene core through a different length linker were synthesized to improve the molecular flexibility. Disappointingly, these compounds demonstrated negligible effects on PARP1 inhibition comparing with compound PA-10 (Table 2). Further, switch of the terminal N substituents to phenyl, afforded new derivatives **15a–e**, showing less improvement in PARP1 activity (Table 3). Therefore, the structural modification of side chain exhibited when n = 1, R1 = R2 = Me, it had best activity. To further explore the impact of core structure, a series of bioisostere was synthesized, compound **19** and **23** was obtained through ibenzo[b,e]oxepin-11(6H)-one (**18**) and dibenzo[b,e]thiepin-11(6H)-one (**22**). Interestingly, both compounds displayed significantly enhanced PARP1 activity and anti-proliferative activity (Table 4), especially compound **19b**, showing an IC_50_ value of 0.75 μM. However, replacing the core structure to anthracen-9(10H)-ylidene or 9H-xanthen- 9-ylidene, led to compounds **26a–c** and **28a–c**, possessing almost no PARP1 inhibitory activity (Table 5). From further analysis of co-crystallization and pharmacophore, we assumed that 2-substituted groups might be an important functional group interacting with PARP1 protein. Therefore, a series of 2-substituted 3-(dimethylamino)propylidene)-6,11-dihydrodibenzo[b,e]oxepin derivatives were obtained from compound **31**. Lots of new compounds displayed significantly enhanced PARP1 activity, especially compound **33e** (hereafter refer to OL-1), showing an IC_50_ value of 0.079 μM against PARP1 and 0.736 μM against MDA-MB-436 cells (Table 6) and being 10-fold more potent than leading compound PA-10, while the one of the positive control Olaparib showing an IC_50_ value of 0.005 μM and 1.12 μM. In addition, we also used the built pharmacophore to estimate the enzymatic activities of all synthesized compounds on PARP1 inhibition. As expected, OL-1 also displayed the best potency on PARP1 inhibition with an estimated IC_50_ value of 0.29 μM (see Table S1). Consequently, based on abovementioned results, OL-1 emerged as the best leading compound with both potent PARP1 inhibition activity and good anti-proliferative effect against MDA-MB-436 cells. Moreover, we used molecular docking to examine the binding states between OL-1 and PARP1. As a result, OL-1 showed a good binding affinity with PARP1 with two hydrogen bonds formed in GLY863 (Fig. 9A). Then, we performed the 10-ns molecular dynamics (MD) simulations on OL-1/PARP1 complex, and obtained the low root-mean-square deviation (RMSD) fluctuations, indicating OL-1 could steadily bind with PARP1 (Fig. 9B). After achieving the most potent compound OL-1, we conducted extensive structure-activity relationship (SAR) studies on part A, B, C (Fig. 10), The activity of the seven membered ring in Part A is superior to six membered ring and when the substituted X is O, the activity is better. In Part B, The more activity is shown when R2 is substituted for different amide groups. In Part C, the carbon chain needs a certain length and the best activity is shown when R1 is substituted by tertiary amine group. This analysis is also consistent with the results of previous molecular docking. Therefore, we selected OL-1 as the parent structure to remain unchanged.

**Table 2:** Inhibition Data of compounds 11 against Recombinant Human PARP1 and MDA-MB-436 cells.


Compound	n	R1	R2	Enzymatic inhibition (IC_50_, μM)^a^	Anti-cell viability (IC_50_, μM)^a^
				PARP1	MDA-MB-436
**11a**	0	Me	H	>20	n.d.^b^
**11b**	0	Me	Me	16.17 ± 1.24	>20
**11c**	1	Me	H	14.21 ± 2.13	>20
**11d (PA-10)**	1	Me	Me	1.65 ± 0.25	5.44 ± 1.01
**11e**	2	Me	H	>20	n.d.^b^
**11f**	2	Me	Me	>20	n.d.^b^

^a^The IC_50_ values are presented as mean ± SD, which is determined by at least three independent experiments.

^b^The n.d. means data are not determined.

**Table 3:** Inhibition Data of compounds 15 against Recombinant Human PARP1 and MDA-MB-436 cells.


Compound	R1	Enzymatic inhibition (IC_50_, μM)^a^	Anti-cell viability (IC_50_, μM)^a^
		PARP1	MDA-MB-436
**15a**	H	>20	n.d.^b^
**15b**	F	>20	n.d.^b^
**15c**	Cl	>20	n.d.^b^
**15d**	Br	>20	n.d.^b^
**15e**	Me	>20	n.d.^b^

^a^The IC_50_ values are presented as mean ± SD, which is determined by at least three independent experiments.

^b^The n.d. means data are not determined.

**Table 4:** Inhibition Data of compounds 19 and 23 against Recombinant Human PARP1 and MDA-MB-436 cells.


Compound	X	n	Enzymatic inhibition (IC_50_, μM)^a^	Anti-cell viability (IC_50_, μM)^a^
			PARP1	MDA-MB-436
**19a**	O	0	13.17 ± 2.33	>20
**19b**	O	1	0.75 ± 0.27	4.14 ± 1.17
**19c**	O	2	>20	n.d.^b^
**23a**	S	0	19.21 ± 3.05	>20
**23b**	S	1	1.04 ± 0.17	17.88 ± 2.11
**23c**	S	2	>20	n.d.^b^

^a^The IC_50_ values are presented as mean ± SD, which is determined by at least three independent experiments.

^b^The n.d. means data are not determined.

**Table 5:** Inhibition Data of compounds 26 and 28 against Recombinant Human PARP1 and MDA-MB-436 cells.


Compound	X	n	Enzymatic inhibition (IC_50_, μM)^a^	Anti-cell viability (IC_50_, μM)^a^
			PARP1	MDA-MB-436
**26a**	CH	0	>20	n.d.^b^
**26b**	CH	1	17.85 ± 2.46	>20
**26c**	CH	2	>20	n.d.^b^
**28a**	O	0	>20	n.d.^b^
**28b**	O	1	>20	n.d.^b^
**28c**	O	2	>20	n.d.^b^

^a^The IC_50_ values are presented as mean ± SD, which is determined by at least three independent experiments.

^b^The n.d. means data are not determined.

**Table 6:** Inhibition Data of compounds 31 and 33 against Recombinant Human PARP1 and MDA-MB-436 cells.


Compound	R1	Enzymatic inhibition (IC_50_, μM)^a^	Anti-cell viability (IC_50_, μM)^a^
		PARP1	MDA-MB-436
**31**	OH	11.24 ± 1.16	>20
**33a**	OMe	19.11 ± 1.67	>20
**33b**	OEt	>20	n.d.^b^
**33c**		>20	n.d.^b^
**33d**	NH_2_	0.344 ± 0.027	1.941 ± 0.514
**33e (OL-1)**		0.079 ± 0.013	0.736 ± 0.223
**33f**		0.724 ± 0.013	1.32 ± 0.31
**33g**		1.26 ± 0.14	4.22 ± 0.75
**33h**		13.47 ± 2.77	19.34 ± 3.11
**33i**		>20	n.d.^b^
**33j**		7.35 ± 1.32	8.23 ± 1.56
**33k**		19.12 ± 2.16	>20
**33l**		4.45 ± 1.29	7.42 ± 0.27
**33m**		18.91 ± 2.29	>20
**33n**		>20	n.d.^b^
**33o**		>20	n.d.^b^
**33p**		>20	n.d.^b^
**PA-10**	—	1.65 ± 0.25	5.44 ± 1.01
**Iniparib**	—	n.d.^b^	14.32 ± 1.56
**Olaparib**	—	0.005 ± 0.001	1.12 ± 0.77

^a^The IC_50_ values are presented as mean ± SD, which is determined by at least three independent experiments.

^b^The n.d. means data are not determined.

### OL-1 induces cell death in breast cancer cells

**Figure 11:**
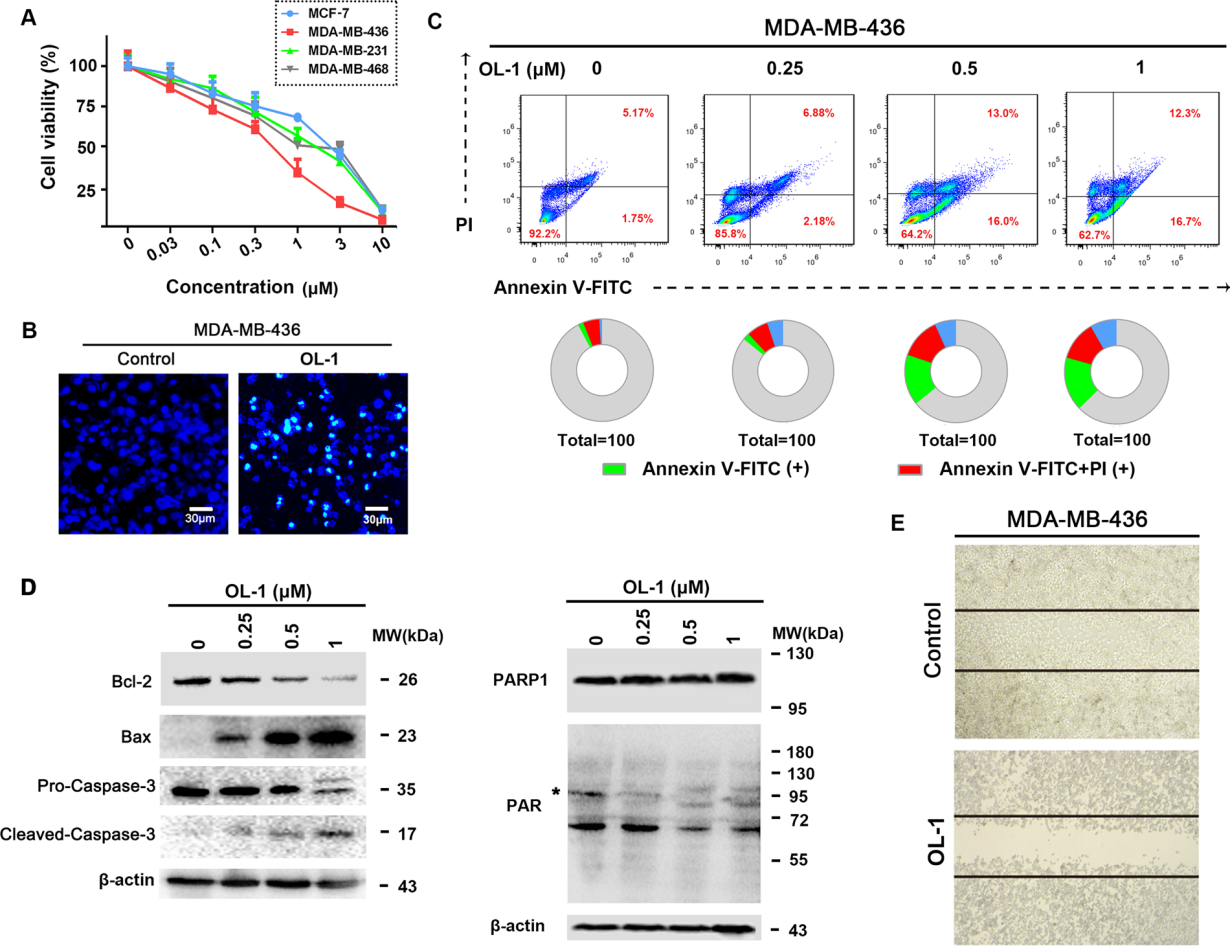
OL-1 induces cell death in breast cancer cells. (**A**) MCF-7, MDA-MB-231, MDA-MB-436, MDA-MB-468 cells were treated with different concentrations of OL-1, and then the cell viabilities were detected by MTT assay. (**B**) Hoechst 33258 fluorescence staining was used to detect DNA breakage. Scale bar = 200 μm. (**C**) MDA-MB-436 cells were treated with 0.25 μM, 0.5 μM and 1 μM OL-1 for 24 h, respectively. After Annexin V-FITC/PI double staining, the apoptosis ratios were analyzed by flow cytometry. (**D**) Western blot analysis of Bax, Bcl-2, Caspase-3, PARP1 and PAR expression levels in OL-1 treated cells. Each lane was loaded with 30 μg cell lysates, β-actin was used as a loading control. (**E**) MDA-MB-436 cells were scratch-wounded by sterile pipette, and then treated with OL-1 for 24 h. Migrated cells were observed by phase-contrast microscope. Scale bar = 100 μm.

To determine the molecular mechanism of OL-1, we firstly found that OL-1 demonstrated anti-proliferative effects against various breast cancer cell lines, especially in the BRCA1 mutant MDA-MB-436 cells (IC_50_ = 5.14 µM) (Fig. 11A). Then we used Hoechst 33258 staining to confirm that OL-1-induced obvious morphologic alterations of apoptosis in MDA-MB-436 cells (Fig. 11B). In addition, we also measured the OL-1-induced apoptotic cell ratio by Annexin V-FITC/PI double staining, which was obviously increased in a concentration-dependent manner (Fig. 11C).

Loss of BRCA1 function leads to genome instability because of defection in DNA repair by homologous recombination^30–32^. Consequently, BRCA1 deficient or mutant cancer cells are commonly sensitized to the inhibition of PARP/PAR-dependent DNA repair mechanisms due to the high-level of DNA damage^33, 34^. Therefore, we determined the effect of OL-1 to inhibit the activation of PARP1 and downstream substrate proteins such as PAR in treated cells by western blot analysis. And we found that OL-1 treatment significantly inhibited the activity of PARP, accompanying with no cleavage of PARP. In addition, the expression of PAR was also decreased in OL-1 treated cells (Fig. 11D). Subsequently, we investigated the involvements of apoptotic markers in OL-1 induced cell death. We found that OL-1 upregulated Bax expression as well as downregulated Bcl-2 expression. And OL-1 treatment also increased cleavage of caspase-3 (Fig. 11D). Moreover, we found that OL-1 could inhibit cell migration of MDA-MB-436 cells (Fig. 11E), indicating OL-1 may inhibit metastasis. These results demonstrate that OL-1 could induce cell apoptosis by inhibiting PARP1 and inhibit cell migration in BRCA1-mutant MDA-MB-436 cells.

### OL-1 displays potent anti-tumor activity *in vivo*

**Figure 12:**
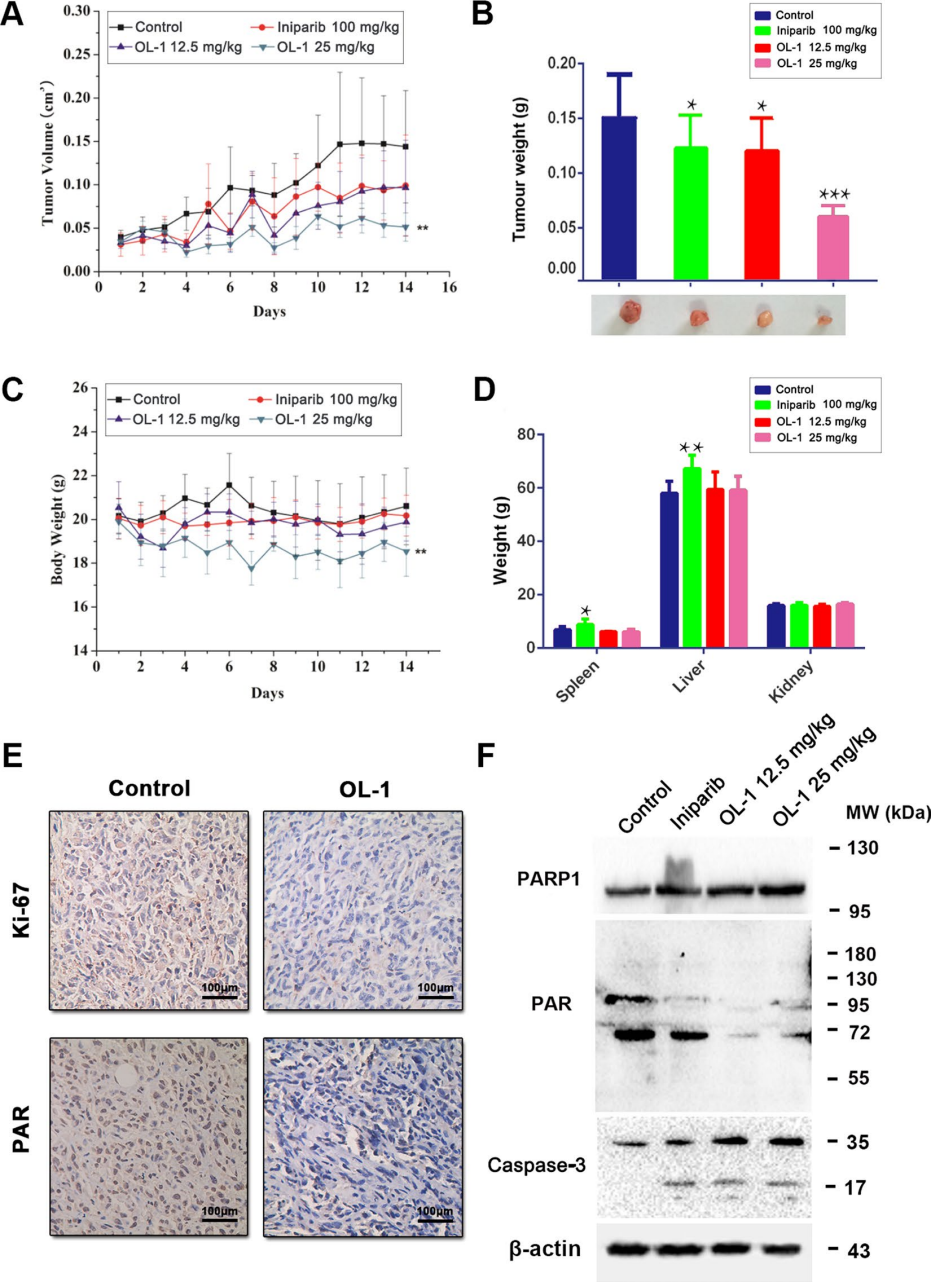
OL-1 displays potent anti-tumor activity *in vivo*. (**A**) Relative tumor volumes of mice (n = 6) injected *i.p.* with PBS, Iniparib (100 mg/kg/d), low dose (12.5 mg/kg/d) and high dose (25 mg/kg/d) of OL-1. **P < 0.01; compared with control group. (**B**) Tumor weights of mice injected *i.p.* PBS, Iniparib (100 mg/kg/d), low dose (12.5 mg/kg/d) and high dose (25 mg/kg/d) of OL-1. *P < 0.05; ***P < 0.001 compared with control group. (**C**) Body weights of mice during treatment. **P < 0.01; compared with control group. (**D**) The weights of liver, spleen and kidney of mice in different groups were measured. *P < 0.05; **P < 0.01; compared with control group. (**E**) Immunohistochemistry analysis of Ki-67 and PAR expression. Scale bar = 200 μm. (**F**) Western blot analysis of PARP, PAR and Caspase-3. Tumor tissues excised from the MDA-MB-436 xenograft mice were lysed. Each lane was loaded with 30 μg cell lysates, β-actin was used as a loading control.

Based upon the anti-proliferative efficacy of OL-1 on MDA-MB-436 cells, we proceeded to assess its efficacy on inhibiting tumor growth in xenograft breast cancer model. After OL-1 treatment, the tumor volumes and tumor weights of high dose group (25 mg/kg/d) were lower than the positive control (Iniparib, 100 mg/kg/d) and low dose groups (12.5 mg/kg/d) (P < 0.05) (Fig. 12A,B). For the toxicity study, compared with the control group and the Iniparib group, high dose of OL-1 (25 mg/kg/d) induced 10.05% loss of body weight during the 14 days of treatment (P < 0.01). In addition, the decrease of body weights in low dose group (12.5 mg/kg/d) was not obvious (Fig. 12C). Meanwhile, liver weights of Iniparib group were significantly decreased (P < 0.01), and spleen weights of mice were also affected by Iniparib (P < 0.05). The liver, spleen and kidney weights were not changed in OL-1 treated groups compared to the Iniparib group (Fig. 12D). In according to the balance between anti-tumor efficacy and toxicity, the low dose (12.5 mg/kg/d) was used as the optimum dose for treatment of tumor growth. To test whether OL-1-induced inhibition of tumor growth *in vivo* was due to reduced cell proliferation, we detected Ki-67 expression in tumor tissues of vehicle- and OL-1-treated mice by immunohistochemical analysis. As a result, OL-1 treatment significantly reduced the positive ratio of Ki-67 compared to the control group (Fig. 12E). For further confirm the mechanism of the therapeutic efficacy of OL-1 *in vivo*, we examined the expression of PARP, PAR and Caspase-3 by western blot analysis. Interestingly, the expression levels of PARP and PAR were highly in accordance with the *in vitro* results (Fig. 12F). Altogether, these results demonstrate that OL-1 displays potent anti-tumor activity *in vivo* by inhibiting PARP1 and its substrate PAR.

## Conclusions

In this study, we have described the discovery and identification of a potent and highly effective PARP1 inhibitor OL-1 (compound **33e**) with a new chemical skeleton. This compound was designed and synthesized based upon co-crystallization studies of a hit compound PA-10. Further in-depth *in vitro* assays were performed with OL-1, which has displayed potent anti-proliferative activities in breast cancer cell lines, especially in MDA-MB-436 cells (*BRAC1* mutation). And PARP enzymatic inhibition assay revealed that OL-1 potently inhibits PARP1 with an IC_50_ value of 0.079 μM. Western blot analysis demonstrated that OL-1 significantly inhibited activities of PARP1 and its downstream substrate PAR. *In vivo* anti-tumor activity assays showed that OL-1 had more potent anti-tumor efficacy than Iniparib in the MDA-MB-436 xenograft model. By the way, OL-1 was also found to inhibit cell migration by *in vitro* would-healing assay, indicating OL-1 may have a potential to inhibit metastasis in triple negative breast cancer. And yet, preliminary pharmacokinetic studies and its efficacy of combination use with other anti-tumor drugs need further intense studies.

## Methods

### Chemistry

All reagents used in this study were purchased from commercial sources without any purification. All ^1^H-NMR and ^13^C-NMR spectra were tested in CDCl_3_ or DMSO-d_6_ by a Bruker-ARX-400 spectrometer. Chemical shifts were recorded in ppm. HRMS data were obtained by LC-ESI-TOF-MS instrument. The melting points were recorded in open capillaries and were uncorrected.

### Molecular docking and molecular dynamics (MD) simulations

Virtual screening of candidate PARP1 inhibitor was processed by the LibDock and CDOCKER modules of Accelrys Discovery Studio (version 3.5)^35,36^. All of the compounds contained in the screening library were downloaded from Drugbank (http://www.drugbank.ca/) and sub-library of ZINC build by NIBS (National Institute of Biological Sciences, Beijing), which contains 33,632 drug-like compounds. Energy minimization of the inhibitors was performed by the CHARMm force field^37^. All residues of PARP1 within 10 Å from the binding site of ligand were defined as the binding sphere. Additionally, Smart Minimizer and CAESAR (Conformer Algorithm based on Energy Screening and Recursive build-up) were applied for *in situ* ligand minimization and generating ligand conformations, respectively. Moreover, in order to detect the binding affinity and complex stability between PARP1 and OL-1, 10 ns MD simulations were processed by GROMACS (version 4.5.5) according to our previous study^38^.

### Protein expression and purification

N-terminal His6 tag was used as purification tag with catalytic domain of human PARP1 residue from 662-1011. Pet28a was used as vector to be produced in **Escherichia coli** BL21 bacteria. The expression of PARP1 protein was induced by 0.4 mM isopropyl β-d-1-thiogalactopyranoside (IPTG). Subsequently, HiTrap Ni^2+^-chelating HP column and HiPrep 26/60 Sephacryl S-300 HR gel-filtration column (GE Healthcare) were used to do the first step purification and following purification, respectively. The purified catPARP1 was stored at −80 °C in a buffer consisting of 140 mM NaCl, 25 mM Tris-HCl, 3 mM KCl pH 7.4.

### Crystallization of PARP1

Crystals of PARP1 were grown at 25 °C using hanging-drop and vapor-diffusion methods. At beginning 9 kits were used for initial crystal screening, including CUBICPHASE1, JCSG+ and PACT (QIAGEN company), as well as CrystalScreen, PEGRX, PEG/ION, INDEX, SALT and SALTRX (Hampton company). Firstly, 1 μL PARP1 (15 mg/mL) was mixed with 1 μL stock solution. After one week crystals could be found from one condition, 2.1 M (NH_4_)_2_SO_4_, 0.1 M Tris-HCl pH 7.2–8.0. When the crystals were reached at a size of 0.002 mm^3^ (0.2 mm × 0.1 mm × 0.1 mm), they were soaked into the well solution containing 5 mM PA-10 and growing condition for overnight. 15% (v/v) glycerol was added as cryoprotectant, and then the crystal was rapidly cooled in liquid nitrogen.

### X-ray diffraction data collection

Nylon loops was used to harvest the soaked PARP1 crystals and then immersed the crystals in mother liquor supplemented with 15% glycerol for 1 min. The synchrotron data were captured on an ADSC Q315 CCD detector (Shanghai Synchrotron Radiation Facility, Shanghai, China). HKL2000 was used to do the data processing.

### Structure solution and refinement

PHASER program was used to do the Molecular replacement a probe PARP1 (Protein Data Bank (PDB) ID 4PJT). Then, REFMAC5 program was used to refine rigid-body by using maximum likelihood. The generated model was manually restructured by COOT program prior to refinement again by REFMAC5 program. PARP1 structure was analyzed by PYMOL program. Refinement statistics details were showed in Table S2.

### Structure-based pharmacophore models construction

Ten co-crystal structure data of PARP1/ligand complex were downloaded from the Protein Data Bank (PDB)^39^. The structure-based pharmacophore models were constructed according to our previous study^40^. In brief, all PARP1/ligand co-crystal structures were turned into a generic reference frame set by using “Multiple Structure Alignment (Modeller)” module in Discovery Studio 3.5. Subsequently, ten individual pharmacophore models based on PARP1/ligand complex were constructed by pharmacophore generation protocol of Discovery Studio 3.5. The identified pharmacophore features were filtered based upon the interaction patterns with PARP1 and showed in Table 1. The generated model was further modified with constraint sphere tolerance by Discovery Studio 3.5 pharmacophore modules.

### PARP1 enzymatic inhibition assays

The PARP1 enzymatic inhibition assay was performed by using Universal Chemiluminescent PARP Assay Kit (Trevigen, Gaithersburg, MD, USA) according to the manufacturer’s instruction and previous report^17^. Briefly, serial dilutions of inhibitor were added to appropriate wells followed by addition of diluted PARP1 enzyme (0.5 Unit/well). After incubation for 10 min at room temperature, distributing 25 μL of 1X PARP Cocktail into each well and incubating the strip wells at room temperature for 60 min. The strip wells were washed with 1X PBS and 0.1% Triton X-100 for twice. Then 50 µL/well of diluted Strep-HRP was added to each well and incubate at room temperature for 60 min. After another wash with PBS, mixing equal volumes of PeroxyGlow™ A and B together and adding 100 µl per well. Finally, immediately taking the chemiluminescent readings. The IC_50_ values of PARP1 inhibitors were determined using Prism 6 software (GraphPad, San Diego, CA, USA).

### Cell culture

Breast cancer cells including MCF-7, MDA-MB-231, MDA-MB-436 and MDA-MB-468 cells were obtained from American Type Culture Collection (ATCC, Manassas, VA, USA). The cells were fed with Leibovitz’s L-15 medium (MDA-MB-231, MDA-MB-436 and MDA-MB-468 cells) or DMEM medium (MCF-7 cells) containing 10% Fetal Bovine Serum, 100 μg/mL streptomycin and 100 U/mL penicillin. MDA-MB-231, MDA-MB-436 and MDA-MB-468 cells were cultured in humidified cell incubator with atmosphere at 37 °C while MCF-7 cells was cultured with 5% CO_2_ at 37 °C.

### Cell viability assay

5 × 10^3^ cells were seeded into each well in 96-well microplates and cultured for 24 h. Then the cells were exposed to different concentrations of OL-1 for 24 h. After drug treatment, the cell viabilities were detected by MTT assay.

### Apoptosis assay

MDA-MB-436 cells (1 × 10^5^ per well) were seeded into 6-well microplates in the presence or absence of OL-1 and cultured for 24 h, then incubated with 500 μL Hoechst 33258 staining solution (0.5 μg/mL) for 30 min at 37 °C. After staining, the apoptotic features were observed under fluorescence microscope. Apoptotic ratio was measured by Annexin-V-FLUOS Staining Kit (Roche, Germany) according to the manufacturer’s protocol followed by flow cytometry (FACS) analysis (Becton Dickinson, Franklin Lakes, NJ).

### Cell migration assay

MDA-MB-436 cells were cultured in 24-well microplates and scratch-wounded by sterilized pipettes. Then the cells were washed with PBS and cultured with normal medium or OL-1. After 24 h incubation, pictures were taken by phase-contrast microscope.

### Western blot analysis

Western blot analysis was carried out briefly as previous description^41^. MDA-MB-436 cells were exposed to OL-1 for indicated time. Both floating cells and adherent were collected. The cell pellets were resuspended with RIPA lysis buffer and PMSF (1 mM) (Beyotime, Haimen, Jiangsu, China) and lysed at 4 °C for 1 h. After 12,000 rpm centrifugation for 10 min, the supernatant was collected to determine the protein content by the BCA Protein Assay Kit (CWBIO, Beijing, China). 30 μg cell lysates in each lane were separated by 8–12% SDS-PAGE and transferred onto PVDF membranes. After pre-blocking in TBST with 5% non-fat milk or BSA for 1 h, the membranes were incubated with primary antibodies overnight at 4 °C, and subsequently incubated with HRP-conjugated secondary antibody at room temperature for 1–2 h. Positive signals were detected by using ECL as the HRP substrate after washing with TBST solution.

### Mouse experiments and *in vivo* xenograft tumor model

All experiments protocols used in this study were carried out in accordance with guidelines of the animal ethics committee (Sichuan University). Thirty-two 6–8 weeks-old female BALB/c nude mice (18–20 g) were subcutaneously injected with MDA-MB-436 cells (1 × 10^7^ cells/mouse). Until the tumor volumes reached 100 mm^3^ (calculated as V = L × W^2^/2), the mice were randomly divided into four groups. Two groups were treated with different doses of OL-1 by *i.p.* (intraperitoneal) injection for 14 days (low dose group, 12.5 mg/kg/d; high dose group, 25 mg/kg/d), whereas the control group was treated with equal amount of normal saline (NS), and the positive drug group was treated with Iniparib, 100 mg/kg/d. Body weight and the tumor size were determined every day until the end of the study. All mice were sacrificed at the end of drug treatment. The organs of mice such as spleen, liver and kidney were harvested and weighed. Tumor tissues were detached and fixed in 4% paraformaldehyde for immunohistochemistry or lysed for western blotting.

### Immunohistochemical analysis

Immunohistochemical analysis was carried out by the method of our previous study^42^. Samples were dehydrated using gradient ethanol, and subsequently paraffin embedded. The paraffin embedded samples were sliced into 5 μm thickness sections. The obtained sections were incubated with primary antibodies against KI-67 and PAR for 15 min followed by biotinylated secondary antibodies and detected with DAB. Nuclei were counterstained with hematoxylin. The numbers of positive cells were counted in at least 6 fields for each section and statistical analyzed.

### Statistical analysis

All the experiments were independently performed by at least three times. The data were statistical analyzed by One-way ANOVA or Student’s t-test of SPSS 17.0 software. All tests with P < 0.05 were considered statistically significant.

## Electronic supplementary material

Supplementary Information
