# Supplementary material for: Crystal structure-based discovery of a novel synthesized PARP1 inhibitor (OL-1) with apoptosis-inducing mechanisms in triple-negative breast cancer
Source: Sci Rep. 2016 Dec 5;6:3. doi: 10.1038/s41598-016-0007-2 (PMC5431371; doi:10.1038/s41598-016-0007-2)
Supplement: Supplementary file 1 — Supplementary Information [file 41598_2016_7_MOESM1_ESM.pdf]

Supplementary information

Crystal structure-based discovery of a novel synthesized PARP1 inhibitor (OL-1) with apoptosis-inducing mechanisms in triple-negative breast cancer

Leilei Fu[1, #], Shuya Wang[1, 2, #], Xuan Wang[1, #], Peiqi Wang[1, 3], Yaxin Zheng[1], Dahong Yao[1], Mingrui Guo[1], Lan Zhang[1, *], Liang Ouyang[1, *]

[1]State Key Laboratory of Biotherapy and Cancer Center, West China Hospital, Sichuan University, and Collaborative Innovation Center of Biotherapy, Chengdu 610041, China

[2]Northwestern University, Feinberg School of Medicine, 303 East Chicago Avenue, Chicago, Illinois 60611, USA

[3]State Key Laboratory of Oral Diseases, West China Hospital of Stomatology, Sichuan University, Chengdu 610041, China

---

[#]These authors contributed equally to this work. *Corresponding author. Tel./Fax: 86-28-85164063. E-mail addresses: zhanglanx_9@126.com (Lan Zhang); ouyangliang@scu.edu.cn (Liang Ouyang).

List of content

**1. Supplementary methods**	**S3-S14**
**2. Table S1 Pharmacophore-based estimated activities of all synthesized compounds on PARP1 enzymatic inhibition**	**S15-S16**
**3. Table S2 Crystallization data and refinement statistics**	**S17**

Supplementary methods

General procedure for the synthesis of compounds 9 (3-brominepropyl triphenylphosphine). To a solution of 1,3-dibromopropane (8.0 g, 39.6 mmol) in xylene (50 mL), triphenylphosphine (10.40 g, 39.6 mmol) was added, and the resulting mixture was warmed to 130 °C for 20 hours, cooled to room temperature and filtered to give the product as white powder[35]. Yield 90%.

General procedure for the synthesis of compound 10 (3-dimethylamino propyl triphenylphosphine). To a solution of **9** (10.0 g, 20 mmol) in ethanol (100 mL), aqueous solution of dimethylamine (20 mL) was added, the mixture was stirred at room temperature for 6 hours. The solvent was removed under reduced pressure to yield the crude product, the crude product was purified by re-crystallization from ethyl alcohol as white crystal plate[36]. Yield 99%.

General procedure for the synthesis of compound 11. To a solution of **10** (1.9 g, 3.7 mmol) in THF (20 mL), a solution of n-BuLi (11.1 mmol) in n-hexane (1.6 N, 7 mL) was added at -10 °C under N_2. The resulting mixture was stirring for 1 hour, and then was added dropwise a solution of **6** (0.31 g, 1.5 mmol) in THF (10 mL), the mixture was allowed to warm up to reflux for 10 hours and cooled to room temperature. The solvent was then removed under reduced pressure, and the resulting crude was dissolved in water (20 mL), extracted with diethyl ether (10 mL) three times. The aqueous layer was acidified to pH=2 with concentrated Hydrochloric acid and extracted with ethyl acetate (15 mL) three times. The aqueous layer was basified with aqueous NaOH (2 N) till pH=7 and removed the solvent to give the desired product as white powder solid.

*2-(10,11-dihydro-5H-dibenzo[a,d][7]annulen-5-ylidene)-N-methylethanamine (**11a**)*.Yield 32%.^{1}H-NMR (DMSO-d_6, 400 MHz) δ_H 7.39-6.99 (8H, m), 5.94 (1H, t, J = 7.6 Hz), 3.97 (3H, s), 3.38 (2H, d, J = 7.6 Hz,), 3.06 (4H, s).^{13}C-NMR (DMSO-d_6, 100 MHz), δ_C 140.4, 139.5, 139.0, 136.2, 130.0, 128.2, 127.4, 126.9, 126.8, 52.5, 36.2, 34.2.MS (ESI), *m/z*250.1 $[M+H]^+$.

*2-(10,11-dihydro-5H-dibenzo[a,d][7]annulen-5-ylidene)-N,N-dimethylethanamine (**11b**)*.Yield 42%.^{1}H-NMR (DMSO-d_6, 400 MHz) δ_H 7.37-6.93 (8H, m), 5.82 (1H, t, J = 7.6 Hz), 3.99 (3H, s), 3.34-3.24 (5H, m), 3.14 (3H, s).^{13}C-NMR (DMSO-d_6, 100 MHz), δ_C 140.7,

139.6, 139.1, 136.7, 130.1, 128.3, 126.9, 126.8, 126.7, 56.8, 44.3, 34.3.MS (ESI), *m/z* 264.2 $[M+H]^+$.

*3-(10,11-dihydro-5H-dibenzo[a,d][7]annulen-5-ylidene)-N-methylpropan-1-amine(**11c**)*.Yield 39%.^{1}H-NMR (DMSO-d_6, 400 MHz) δ_H 7.45-6.87 (8H, m), 6.00 (1H, t, *J* = 7.4 Hz), 3.26 (3H, s), 3.05 (4H, s), 2.63-2.53 (2H, m), 2.33-1.95 (2H, m).^{13}C-NMR (DMSO-d_6, 100 MHz), δ_C 140.4, 139.5, 139.1, 135.3, 130.2, 128.2, 126.9, 126.8, 126.7, 48.6, 36.7, 34.2, 28.6.MS (ESI), *m/z* 264.2 $[M+H]^+$.

*3-(10,11-dihydro-5H-dibenzo[a,d][7]annulen-5-ylidene)-N,N-dimethylpropan-1-amine(**11d**)*.Yield 46%.^{1}H-NMR (DMSO-d_6, 400 MHz) δ_H 7.45-6.91 (8H, m), 5.99 (1H, t, *J* = 8.4 Hz,), 3.41 (2H, t, *J* = 15.0 Hz,), 3.05 (4H, s), 2.84 (6H, s,), 2.24-2.13(2H, m).^{13}C-NMR (DMSO-d_6, 100 MHz),δ_C 140.2, 139.6, 138.9, 135.0, 130.0, 128.3, 127.2, 126.9, 126.8, 58.1, 45.5, 34.3, 28.9.MS (ESI), *m/z* 278.2 $[M+H]^+$.

*4-(10,11-dihydro-5H-dibenzo[a,d][7]annulen-5-ylidene)-N-methylbutan-1-amine (**11e**)*.Yield 48%. ^{1}H-NMR (DMSO-d6, 400 MHz) δ_H7.50-6.85 (8H, m), 5.92 (1H, t, *J*= 6.4 Hz), 3.26 (3H, s), 3.05 (4H, s), 2.57 (2H, t, *J* = 7.2 Hz), 2.02-1.93 (2H, m), 1.78-1.34 (2H, m).^{13}C-NMR (DMSO-d_6, 100 MHz), δ_C 140.4, 139.5, 139.1, 136.3, 130.1, 128.2, 128.0, 126.7, 126.5, 50.9, 36.7, 35.2, 28.9, 27.6.MS (ESI), *m/z* 278.3 $[M+H]^+$.

*4-(10,11-dihydro-5H-dibenzo[a,d][7]annulen-5-ylidene)-N,N-dimethylbutan-1-amine (**11f**)*.Yield 45%.^{1}H-NMR (DMSO-d_6, 400 MHz) δ_H 7.46-6.46 (8H, m), 5.99 (1H, t, *J* = 6.4 Hz,), 3.27-2.80 (6H, m), 2.28-1.76 (8H, m), 1.70-1.27 (2H, m).^{13}C-NMR (DMSO-d_6, 100 MHz),δ_C 141.4, 139.6, 139.2, 136.3, 130.7, 128.2, 128.0, 126.9, 126.8, 59.0, 45.5, 34.2, 27.4, 25.6.MS (ESI), *m/z* 292.2 $[M+H]^+$.

General procedure for the synthesis of compound 15. To a solution of **6** (0.31 g, 1.5 mmol) in THF (10 mL), a solution of Grignard reagent **14** (3 mmol) in THF (10 mL) was added at -10 °C under N_2. The mixture was allowed to warm up to room temperature for 10 hours. The solvent was then removed under reduced pressure, and the resulting crude was dissolved in water (20 mL), extracted with diethyl ether (10 mL) three times. The aqueous layer was acidified to pH=2 with concentrated Hydrochloric acid and extracted with ethyl acetate (15 mL) three times. The crude product was purified by silica gel chromatography eluted with CH_2Cl_2:CH_3OH=10:1.

5-benzylidene-10,11-dihydro-5H-dibenzo[a,d][7]annulene (***15a***).Yield 76%,^{1}H-NMR ($CDCl_3$, 400 MHz), δ_H 7.50 (1H, m), 7.00-7.30 (12H, m), 6.81(1H, s), 3.46-3.38 (2H, m), 2.98-2.74 (2H, m); ^{13}C-NMR ($CDCl_3$, 100 MHz), δ_C 143.8, 141.2., 140.5, 139.1, 137.6, 137.2, 130.2, 129.9, 129.2, 129.2, 128.4, 128.3, 128.0, 127.9, 127.9, 127.7, 127.4, 126.7, 126.4, 126.2, 33.6, 32.1.MS (ESI), *m/z* 283.2 $[M+H]^+$.

5-(4-chlorobenzylidene)-10,11-dihydro-5H-dibenzo[a,d][7]annulene (***15b***).Yield 65%, ^{1}H-NMR ($CDCl_3$, 400 MHz), δ_H 7.48 (1H, m), 7.30-6.94 (11H, m), 6.76(1H, s), 3.44-3.39 (2H, m), 2.91-2.81 (2H, m); ^{13}C-NMR ($CDCl_3$, 100 MHz), δ_C 144.6, 141.5., 140.1, 139.1, 137.6, 135.7, 132.3, 130.4, 130.4, 130.2, 128.5, 128.4, 128.3, 128.2, 128.2, 127.9, 127.8, 127.5, 126.5, 126.2, 33.6, 32.1.MS (ESI), *m/z*318.1 $[M+H]^+$.

5-(4-bromobenzylidene)-10,11-dihydro-5H-dibenzo[a,d][7]annulene (***15c***).Yield 57%, ^{1}H-NMR ($CDCl_3$, 400 MHz), δ_H 7.48 (1H, m), 7.30-6.94 (11H, m), 6.76(1H, s), 3.46-3.31 (2H, m), 2.91-2.79 (2H, m); ^{13}C-NMR ($CDCl_3$, 100 MHz), δ_C 144.7, 141.5., 140.0, 139.0, 137.6, 136.1, 131.1, 131.1, 130.7, 130.7, 130.2, 128.6, 128.5, 128.1, 128.0, 127.9, 127.6, 126.6, 126.2, 120.5, 33.6, 32.1.MS (ESI), *m/z*363.2 $[M+H]^+$.

5-(4-fluorobenzylidene)-10,11-dihydro-5H-dibenzo[a,d][7]annulene (***15d***).Yield 69%, ^{1}H-NMR ($CDCl_3$, 400 MHz), δ_H 7.48 (1H, m), 7.30-6.96 (10H, m), 6.84 (2H, t, J = 8.8 Hz), 6.76 (1H, s), 3.47-3.33 (2H,m), 2.92-2.84 (2H, m); ^{13}C-NMR ($CDCl_3$, 100 MHz), δ_C 144.6, 141.5., 140.1, 139.1, 137.6, 135.7, 132.3, 130.4, 130.4, 130.2, 128.5, 128.4, 128.3, 128.2, 128.2, 127.9, 127.8, 127.5, 126.5, 126.2, 33.6, 32.1.MS (ESI), *m/z*302.3 $[M+H]^+$.

5-(4-methylbenzylidene)-10,11-dihydro-5H-dibenzo[a,d][7]annulene (***15e***).Yield 70%, ^{1}H-NMR ($CDCl_3$, 400 MHz), δ_H 7.48 (1H, m), 7.30-6.92 (11H, m), 6.80 (1H, s), 3.46-3.25 (2H,m), 2.98-2.88 (2H, m), 2.30 (3H, s); ^{13}C-NMR ($CDCl_3$, 100 MHz), δ_C 142.8, 142.0, 140.7, 139.1, 137.6, 136.5, 134.3, 130.2, 129.8, 129.2, 129.2, 129.0, 128.7, 128.3, 128.3, 128.0, 127.7, 127.3, 126.4, 126.1, 33.6, 32.1, 21.2.MS (ESI), *m/z*297.2 $[M+H]^+$.

General procedure for the synthesis of compound 19. To a solution of **10** (1.9 g, 3.7 mmol) in THF (20 mL), a solution of n-BuLi (11.1 mmol) in n-hexane (1.6 N, 7 mL) was added at -10 °C under N_2. The resulting mixture was stirring for 1 hour, and then was added dropwise a solution of **18** (0.32 g, 1.5 mmol) in THF (10 mL), the mixture was allowed to warm up to reflux for 10 hours and cooled to room temperature. The solvent was then

removed under reduced pressure, and the resulting crude was dissolved in water (20 mL), extracted with diethyl ether (10 mL) three times. The aqueous layer was acidified to pH=2 with concentrated Hydrochloric acid and extracted with ethyl acetate (15 mL) three times. The aqueous layer was basified with aqueous NaOH (2 N) till pH=7 and removed the solvent to give the desired product, and the crude product was purified by silica gel chromatography eluted with CH_2Cl_2:CH_3OH=10:1.

*2-(dibenzo[b,e]oxepin-11(6H)-ylidene)-N,N-dimethylethanamine (**19a**)*.Yield 70%, ^{1}H-NMR ($CDCl_3$, 400 MHz), δ_H 7.50-7.07 (6H, m), 6.97 (1H, dd, J = 8.4, 2.2 Hz), 6.78 (1H, dd, J = 8.8, 2.4 Hz), 5.91 (1H , t, J = 7.6 Hz,), 5.43 (br s, 2H), 3.02 (2H, d, J = 7.2 Hz), 2.75 (br s, 6H). ^{13}C-NMR ($CDCl_3$, 100 MHz), δ_C 156.1, 141.6, 139.7, 137.7, 133.2, 129.9, 129.4, 128.8, 127.9, 127.7, 127.3, 126.7, 122.6, 116.4, 71.1, 56.9, 44.4.MS (ESI), *m/z* 267.5[M+H]$^+$.

*3-(dibenzo[b,e]oxepin-11(6H)-ylidene)-N,N-dimethylpropan-1-amine (**19b**)*. Yield 62%.^{1}H-NMR ($CDCl_3$, 400 MHz), δ_H 7.48-7.02 (6H, m), 6.93 (1H, dd, J = 8.4, 2.2 Hz,), 6.78 (1H, dd, J = 8.4, 2.2 Hz,), 6.02 (1H, t, J = 7.6 Hz,), 5.43 (brs, 2H), 3.41 (2H, t, J = 7.2 Hz,), 2.84 (brs, 6H), 2.27-2.19 (2H, m). ^{13}C-NMR ($CDCl_3$, 100 MHz), δ_C 156.2, 141.4, 139.6, 133.8, 133.1, 130.4, 129.7, 128.5, 127.6, 127.6, 127.2, 126.4, 122.3, 116.9, 72.1, 58.12, 45.6, 28.8.MS (ESI), *m/z* 280.2[M+H]$^+$.

*4-(dibenzo[b,e]oxepin-11(6H)-ylidene)-N,N-dimethylbutan-1-amine (**19c**)*.Yield 55%. ^{1}H-NMR ($CDCl_3$, 400 MHz), δ_H 7.50-7.04 (6H, m), 6.93-6.89 (1H, m), 6.77 (1H, dd, J = 8.4, 2.2 Hz), 6.01 (1H, t, J = 7.6 Hz), 5.42 (s, 2H), 3.03 (2H, t, J = 7.6 Hz), 2.19-1.89 (8H, m), 1.70-1.40 (2H, m). ^{13}C-NMR ($CDCl_3$, 100 MHz), δ_C 155.9, 141.5, 139.7, 137.5, 133.2, 132.8, 129.7, 128.7, 127.9, 127.7, 127.4, 126.7, 122.6, 116.9, 71.0, 59.1, 45.6, 27.4, 25.6.MS (ESI), *m/z* 294.2[M+H]$^+$.

General procedure for the synthesis of compound 23. To a solution of **10** (1.9 g, 3.7 mmol) in THF (20 mL), a solution of n-BuLi (11.1 mmol) in n-hexane (1.6 N, 7 mL) was added at -10 °C under N_2. The resulting mixture was stirring for 1 hour, and then was added dropwise a solution of **22** (0.34 g, 1.5 mmol) in THF (10 mL), the mixture was allowed to warm up to reflux for 10 hours and cooled to room temperature. The solvent was then removed under reduced pressure, and the resulting crude was dissolved in water (20 mL),

extracted with diethyl ether (10 mL) three times. The aqueous layer was acidified to pH=2 with concentrated Hydrochloric acid and extracted with ethyl acetate (15 mL) three times. The aqueous layer was basified with aqueous NaOH (2 N) till pH=7 and removed the solvent to give the desired product, and the crude product was purified by silica gel chromatography eluted with CH_2Cl_2:CH_3OH=10:1.

*2-(dibenzo[b,e]thiepin-11(6H)-ylidene)-N,N-dimethylethanamine (**23a**)*.Yield 45%. ^{1}H-NMR ($CDCl_3$, 400 MHz), δ_H 7.64-7.12 (7H, m), 7.08-6.94 (1H, m), 5.91 (1H, t, J = 7.2 Hz), 4.66 (2H, s), 3.02 (2H, d, J = 7.2 Hz), 2.75 (6H, s). ^{13}C-NMR ($CDCl_3$, 100 MHz), δ_C 144.3, 138.4, 136.3, 135.3, 134.4, 129.5, 128.8, 128.6, 127.5, 127.4, 127.2, 126.8, 126.7, 125.5, 56.9, 44.4, 33.3.MS (ESI), *m/z* 282.7[M+H]$^+$.

*3-(dibenzo[b,e]thiepin-11(6H)-ylidene)-N,N-dimethylpropan-1-amine (**23b**)*.Yield 35%. ^{1}H-NMR ($CDCl_3$, 400 MHz), δ_H7.59-6.90 (8H, m), 6.01 (1H, t, J = 7.2 Hz), 4.66 (s, 2H), 3.41 (t, J = 7.2 Hz, 2H), 2.84 (s, 6H), 2.24 (td, J = 14.7, 12.4 Hz, 2H). ^{13}C-NMR ($CDCl_3$, 100 MHz), δ_C 144.5, 138.2, 136.6, 136.3, 135.2, 129.8, 129.6, 128.9, 128.5, 127.4, 127.2, 126.7, 126.6, 125.5, 58.1, 45.6, 33.4, 28.7.MS (ESI), *m/z* 296.4[M+H]$^+$.

*4-(dibenzo[b,e]thiepin-11(6H)-ylidene)-N,N-dimethylbutan-1-amine (**23c**)*.Yield 34%. ^{1}H-NMR ($CDCl_3$, 400 MHz), δ_H 7.61-7.01(8H, m), 5.91 (1H, t, J = 7.2 Hz,), 4.64 (2H, s), 3.03 (2H, t, J = 7.2 Hz), 2.30-1.84 (8H, m), 1.72-1.35 (2H, m). ^{13}C NMR ($CDCl_3$, 100 MHz), δ_C 144.3, 138.4, 136.4, 136.3, 135.3, 130.8, 129.6, 128.7, 128.6, 127.4, 127.3, 126.7, 126.6, 125.4, 59.0, 45.5, 33.3, 27.5, 25.6.MS (ESI), *m/z* 310.3[M+H]$^+$.

General procedure for the synthesis of compound 26. To a solution of **10** (1.9 g, 3.7 mmol) in THF (20 mL), a solution of n-BuLi (11.1 mmol) in n-hexane (1.6 N, 7 mL) was added at -10 °C under N_2. The resulting mixture was stirring for 1 hour, and then was added dropwise a solution of **25** (0.29 g, 1.5 mmol) in THF (10 mL), the mixture was allowed to warm up to reflux for 10 hours and cooled to room temperature. The solvent was then removed under reduced pressure, and the resulting crude was dissolved in water (20 mL), extracted with diethyl ether (10 mL) three times. The aqueous layer was acidified to pH=2 with concentrated Hydrochloric acid and extracted with ethyl acetate (15 mL) three times. The aqueous layer was basified with aqueous NaOH (2 N) till pH=7 and removed the solvent to give the desired product, and the crude product was purified by silica gel

chromatography eluted with CH_2Cl_2:CH_3OH=10:1.

2-(anthracen-9(10H)-ylidene)-N,N-dimethylethanamine (***26a***). Yield 35%. ^{1}H-NMR ($CDCl_3$, 400 MHz), δ_H 7.46-6.97 (8H, m), 6.16 (1H, t, *J* = 7.2 Hz,), 4.35 (2H, s), 3.02 (2H, d, *J* = 7.2 Hz,), 2.75 (6H s). ^{13}C-NMR ($CDCl_3$, 100 MHz), δ_C 134.6, 134.2, 133.9, 131.5, 126.4, 125.5, 125.2, 122.2, 120.3, 56.9, 44.4, 39.0.MS (ESI), *m/z* 250.5$[M+H]^+$.

3-(anthracen-9(10H)-ylidene)-N,N-dimethylpropan-1-amine (***26b***)**.** Yield 41%. ^{1}H-NMR ($CDCl_3$, 400 MHz), δ_H7.50-6.87 (8H, m), 6.22 (1H, t, *J* = 7.2 Hz,), 4.34 (2H, s), 3.40 (2H, t, *J* = 7.4 Hz), 2.83 (6H, s), 2.34-2.29 (2H, m). ^{13}C-NMR ($CDCl_3$, 100 MHz), δ_C 134.9, 134.0, 133.7, 131.7, 126.3, 125.6, 125.4, 122.1, 122.0, 58.1, 45.6, 39.0, 28.7.MS (ESI), *m/z* 264.2$[M+H]^+$.

4-(anthracen-9(10H)-ylidene)-N,N-dimethylbutan-1-amine (***26c***).Yield 33%. ^{1}H-NMR ($CDCl_3$, 400 MHz), δ_H 7.53-6.90 (8H, m), 6.20 (1H, t, *J* = 6.4 Hz), 4.31 (2H, s), 3.04 (2H, t, *J* = 6.4 Hz), 2.26-1.86 (8H, m), 1.76-1.32 (2H, m). ^{13}C-NMR ($CDCl_3$, 100 MHz), δ_C 134.6, 134.0, 133.8, 131.5, 126.4, 125.6, 125.4, 122.1, 122.0, 59.1, 45.6, 39.0, 27.5, 25.6.MS (ESI), *m/z* 278.2$[M+H]^+$.

General procedure for the synthesis of compound 28. To a solution of **10** (1.9 g, 3.7 mmol) in THF (20 mL), a solution of n-BuLi (11.1 mmol) in n-hexane (1.6 N, 7 mL) was added at -10 °C under N_2. The resulting mixture was stirring for 1 hour, and then was added dropwise a solution of **27** (0.29 g, 1.5 mmol) in THF (10 mL), the mixture was allowed to warm up to reflux for 10 hours and cooled to room temperature. The solvent was then removed under reduced pressure, and the resulting crude was dissolved in water (20 mL), extracted with diethyl ether (10 mL) three times. The aqueous layer was acidified to pH=2 with concentrated Hydrochloric acid and extracted with ethyl acetate (15 mL) three times. The aqueous layer was basified with aqueous NaOH (2 N) till pH=7 and removed the solvent to give the desired product, and the crude product was purified by silica gel chromatography eluted with CH_2Cl_2:CH_3OH=10:1.

N,N-dimethyl-2-(9H-xanthen-9-ylidene)ethanamine (***28a***).Yield 33%. ^{1}H-NMR ($CDCl_3$, 400 MHz), δ_H 7.46-7.39 (2H, m), 7.35-7.10 (4H, m), 6.99 (2H, td, *J* = 8.4, 2.2 Hz), 6.12 (1H, t, *J* = 7.2 Hz), 3.02 (2H, d, *J* = 7.2 Hz,), 2.75 (s, 6H). ^{13}C-NMR ($CDCl_3$, 100 MHz), δ_C 150.6, 132.4, 128.3, 125.5, 125.4, 123.5, 122.9, 122.6, 117.4, 56.9, 44.4.MS (ESI), *m/z*

252.6[M+H]$^+$.

N,N-dimethyl-3-(9H-xanthen-9-ylidene)propan-1-amine ***(28b)***.Yield 33%.^{1}H-NMR ($CDCl_3$, 400 MHz), δ_H7.50-7.40 (2H,m), 7.34-7.15 (4H, m), 6.92 (2H, td, *J* = 7.4, 2.2 Hz,), 6.18 (1H, t, *J* = 6.4 Hz,), 3.41 (2H, t, *J* = 6.4 Hz,), 2.84 (s, 6H), 2.48-2.29 (2H, m). ^{13}C-NMR ($CDCl_3$, 100 MHz), δ_C 151.6, 129.0, 128.2, 125.9, 125.2, 123.4, 122.9, 122.5, 117.3, 58.2, 45.6, 28.8.MS (ESI), *m/z* 266.1[M+H]$^+$.

N,N-dimethyl-4-(9H-xanthen-9-ylidene)butan-1-amine ***(28c)***.Yield 51%.^{1}H-NMR ($CDCl_3$, 400 MHz), δ_H 7.51-7.41 (2H, m), 7.37-7.12 (4H, m), 6.94 (2H, td, *J* = 8.4, 2.2 Hz), 6.22 (1H, t, *J* = 7.2 Hz,), 3.04 (2H, t, *J* = 7.2Hz), 2.28-1.79 (8H, m), 1.78-1.40 (2H, m). ^{13}C-NMR ($CDCl_3$, 100 MHz), δ_C 150.6, 129.3, 128.3, 128.1, 125.4, 123.5, 122.9, 122.6, 117.4, 59.0, 45.6, 27.5, 25.6.MS (ESI), *m/z* 280.3[M+H]$^+$.

General procedure for the synthesis of compound 30 (2-(11-oxo-6,11-dihydrodibenzo[b,e] oxepin-2-yl)acetic acid). To a solution of 2-(11-oxo-6,11-dihydrodibenzo[b,e]oxepin-2-yl)acetic acid(5.0 g, 17.5 mmol) and 85% phosphoric acid (1.8 mmol) in toluene (50 mL), acetylchloride (1.2 eq) was added, and the resulting mixture was allowed to warmed up to 100 °C for 10 hours. Activated carbon (500 mg) was added and stirred 1 hours at 100 °C, the mixture was filtered and cooled to room temperature to give the crude product. The crude product was purified by re-crystallization from ethyl acetate and n-hexane as slight yellow solid. Yield 70%. ^{1}H-NMR ($CDCl_3$, 400 MHz), δ_H 8.12 (1H, d, *J* = 2.3 Hz), 7.88 (1H, dd, *J* = 7.6, 1.1 Hz), 7.55 (1H, td, *J* = 7.4, 1.3 Hz), 7.46 (1H, td, *J* = 7.6, 1.1 Hz), 7.42 (1H, dd, *J* = 8.4, 2.3 Hz), 7.36 (1H, d, *J* = 7.4 Hz), 7.03 (1H, d, *J* = 8.4 Hz), 5.18 (2H, s), 3.67 (2H, s); ^{13}C-NMR ($CDCl_3$, 100 MHz), δ_C 190.9, 177.2, 160.6, 140.4, 136.4, 135.5, 132.8, 132.6, 129.5, 129.3, 127.8, 127.1, 125.2, 121.2, 73.6, 39.9.

General procedure for the synthesis of compound 31 (2-(11-(3-(dimethylamino)propylidene) -6,11-dihydrodibenzo[b,e]oxepin-2-yl)acetic acid). To a solution of **10** (1.9 g, 3.7 mmol) in THF (20 mL), a solution of n-BuLi (11.1 mmol) in n-hexane (1.6 N, 7 mL) was added at -10 °C under N_2. The resulting mixture was stirring for 1 hour, and then was added dropwise a solution of **30** (0.4 g, 1.5 mmol) in THF (10 mL), the mixture was allowed to warm up to reflux for 10 hours and cooled to room temperature.

The solvent was then removed under reduced pressure, and the resulting crude was dissolved in water (20 mL), extracted with diethyl ether (10 mL) three times. The aqueous layer was acidified to pH=2 with concentrated Hydrochloric acid and extracted with ethyl acetate (15 mL) three times. The aqueous layer was basified with aqueous NaOH (2 N) till pH=7 and removed the solvent to give the desired product as white powder solid. Yield 30%. ^{1}H-NMR (DMSO-d$_6$, 400 MHz), δ_H 12.3 (1H, br s), 7.25-7.40 (4H, m), 7.06 (1H, d, J = 2.2 Hz), 7.04 (1H, dd, J = 8.1, 2.2 Hz), 6.78 (1H, d, J = 8.1 Hz), 5.65 (1H, t, J = 7.2 Hz), 5.12 (2H, br s), 3.56 (2H, s), 3.25 (2H, t, J = 7.7 Hz), 2.79 (2H, q, J = 7.4 Hz), 2.72 (6H, br s); ^{13}C-NMR ($CDCl_3$, 100 MHz), δ_C 173.4, 154.3, 145.0, 141.4, 133.9, 132.3, 131.1, 129.6, 128.2, 128.2, 127.7, 127.3, 126.4, 123.3, 119.7, 69.9, 55.9, 42.2, 42.2, 40.0, 24.9.

General procedure for the synthesis of compounds 33a-o. To a solution of **31** (338 mg, 1 mmol) in dichloromethane (20 mL), thionyl chloride (238 mg, 2 mmol) was added at temperature for 10 hours, the solvent was removed under reduce pressure and the resulting was diluted with dichloromethane (5 mL) and added to a solution of appropriate amine and Et_3N dropwise. The organic layer was washed with water and brine and dried over anhydrous sodium sulfate, the solvent was removed under reduce pressure to give the crude product, purified by silica gel chromatography eluted with CH_2Cl_2:CH_3OH=10:1.

*Methyl 2-(11-(3-(dimethylamino)propylidene)-6,11-dihydrodibenzo[b,e]oxepin-2-yl)acetate (**33a**)*. Yield 72%. ^{1}H-NMR ($CDCl_3$, 400 MHz), δ_H 7.20-7.40 (4H, m), 7.06 (1H, d, J = 2.2 Hz), 7.04 (1H, dd, J = 8.3, 2.2 Hz), 6.83 (1H, d, J = 8.3 Hz), 5.72 (1H, t, J = 7.2 Hz), 5.43 (2H, br s), 3.67 (1H, s), 3.45 (3H, s), 2.56 (2H, m), 2.45 (2H, t, J = 7.2 Hz), 2.22 (6H, br s); ^{13}C-NMR ($CDCl_3$, 100 MHz) δ_C 172.2, 154.6, 145.6, 139.7, 133.7, 132.0, 130.7, 130.0, 129.1, 127.5, 127.5, 126.3, 125.7, 123.9, 119.8, 70.4, 59.5, 53.4, 52.0, 45.4, 40.2, 28.1.MS (ESI), *m/z*352.2[M+H]$^+$.

*Ethyl 2-(11-(3-(dimethylamino)propylidene)-6,11-dihydrodibenzo[b,e]oxepin-2-yl)acetate (**33b**)*. Yield 64%. ^{1}H-NMR ($CDCl_3$, 400 MHz), δ_H 7.20-7.40 (4H, m), 7.06 (1H, d, J = 2.2 Hz), 7.04 (1H, dd, J = 8.3, 2.2 Hz), 6.83 (1H, d, J = 8.3 Hz), 5.72 (1H, t, J = 7.2 Hz), 5.43 (2H, br s), 4.13 (1H, q, J = 7.2 Hz), 3.45 (2H, s), 2.56 (2H, m), 2.45 (2H, t, J = 7.2 Hz), 2.22 (6H, br s), 1.24 (3H, t, J = 7.2 Hz); ^{13}C-NMR ($CDCl_3$, 100 MHz) δ_C 171.8, 154.6, 145.5, 140.0, 133.6, 132.0, 130.2, 130.0, 129.1, 127.5, 127.4, 126.3, 126.0, 123.9,

119.8, 70.4, 60.8, 59.2, 45.9, 45.1, 40.4, 27.7, 14.2.MS (ESI), *m/z*366.4[M+H]$^+$.

*Isopropyl 2-(11-(3-(dimethylamino)propylidene)-6,11-dihydrodibenzo[b,e]oxepin-2-yl)acetate (**33c**).*^{1}H-NMR ($CDCl_3$, 400 MHz), δ_H 7.20-7.40 (4H, m), 7.06 (1H, d, *J* = 2.2 Hz), 7.04 (1H, dd, *J* = 8.3, 2.2 Hz), 6.83 (1H, d, *J* = 8.3 Hz), 5.72 (1H, t, *J* = 7.2 Hz), 5.43 (2H, br s), 4.98 (1H, m), 3.45 (2H, s), 2.56 (2H, m), 2.45 (2H, t, *J* = 7.2 Hz), 2.22 (6H, br s), 1.21 (6H, d, *J* = 6.4 Hz). ^{13}C-NMR ($CDCl_3$, 100 MHz) δ_C 171.8, 154.6, 145.5, 140.0, 133.6, 132.0, 130.2, 130.0, 129.1, 127.5, 127.4, 126.3, 126.0, 123.9, 119.8, 70.4, 68.1, 59.4, 45.3, 45.3, 40.8, 27.9, 21.8, 21.8.MS (ESI), *m/z*380.1[M+H]$^+$.

*2-(11-(3-(dimethylamino)propylidene)-6,11-dihydrodibenzo[b,e]oxepin-2-yl)acetamide (**33d**)*.Yield 62%.^{1}H-NMR ($CDCl_3$, 400 MHz), δ_H 7.20-7.40 (4H, m), 7.06 (1H, d, *J* = 2.2 Hz), 7.04 (1H, dd, *J* = 8.3, 2.2 Hz), 6.83 (1H, d, *J* = 8.3 Hz), 5.72 (1H, t, *J* = 7.2 Hz), 5.58 (2H, br s), 3.45 (2H, s), 2.56 (2H, m), 2.45 (2H, t, *J* = 7.2 Hz), 2.22 (6H, br s); ^{13}C-NMR ($CDCl_3$, 100 MHz), δ_C 173.6, 154.8, 145.5, 139.6, 133.6, 132.2, 130.9, 130.0, 129.2, 127.6, 127.5, 126.6, 126.3, 124.2, 120.3, 70.4, 59.4, 45.4, 45.4, 42.5, 28.3.MS (ESI), *m/z*337.8[M+H]$^+$.

*2-(11-(3-(dimethylamino)propylidene)-6,11-dihydrodibenzo[b,e]oxepin-2-yl)acetohydrazide (**33e**)*.Yield 77%.^{1}H-NMR ($CDCl_3$, 400 MHz), δ_H 7.51-7.16 (4H, m), 7.08 (1H, d, *J* = 2.2 Hz), 7.02 (1H, dd, *J* = 8.3, 2.2 Hz, 1H), 6.88 (1H, d, *J* = 8.3 Hz), 5.96 (1H , t, *J* =7.2 Hz), 5.43 (2H, brs,), 3.63 (2H, s), 3.41 (2H,m), 2.34 (2H,t, *J* = 7.2 Hz), 2.23 (6H,br s); ^{13}C-NMR ($CDCl_3$, 100 MHz) , δ_C 172.4, 152.1, 144.8, 139.7, 133.7, 130.9, 130.6, 129.9, 129.6, 128.7, 128.7, 127.9, 127.2, 126.7, 117.8, 71.2, 58.2, 45.6, 39.9, 28.8.MS (ESI), *m/z*352.7[M+H]$^+$.

*2-(11-(3-(dimethylamino)propylidene)-6,11-dihydrodibenzo[b,e]oxepin-2-yl)-N-methylacetamide (**33f**)*.Yield 65%.^{1}H-NMR ($CDCl_3$, 400 MHz), δ_H 7.20-7.40 (4H, m), 7.06 (1H, d, *J* = 2.2 Hz), 7.04 (1H, dd, *J* = 8.3, 2.2 Hz), 6.83 (1H, d, *J* = 8.3 Hz), 5.72 (1H, t, *J* = 7.2 Hz), 5.49 (2H, br s), 3.48 (2H, s), 3.18 (2H, q, *J* = 6.9 Hz), 2.73 (3H, d, *J* = 4.8 Hz), 2.56 (2H, m), 2.45 (2H, t, *J* = 7.2 Hz), 2.22 (6H, br s); ^{13}C-NMR ($CDCl_3$, 100 MHz) δ_C 170.4, 154.7, 145.6, 139.7, 133.6, 132.3, 130.8, 130.0, 129.2, 127.6, 127.5, 126.7, 126.3, 124.1, 120.2, 70.4, 59.4, 45.4, 45.4, 42.8, 28.3, 26.5.MS (ESI), *m/z*351.2[M+H]$^+$.

*2-(11-(3-(dimethylamino)propylidene)-6,11-dihydrodibenzo[b,e]oxepin-2-yl)-N-ethylacetamide (**33g**)*.Yield 70%^{1}H-NMR ($CDCl_3$, 400 MHz), δ_H 7.20-7.40 (4H, m), 7.06 (1H,

d, *J* = 2.2 Hz), 7.04 (1H, dd, *J* = 8.3, 2.2 Hz), 6.83 (1H, d, *J* = 8.3 Hz), 5.72 (1H, t, *J* = 7.2 Hz), 5.43 (2H, br s), 3.45 (2H, s), 3.22 (2H, m), 2.56 (2H, m), 2.45 (2H, t, *J* = 7.2 Hz), 2.22 (6H, br s), 1.04 (3H, t, *J* = 7.2 Hz); ^{13}C-NMR ($CDCl_3$, 100 MHz) δ_C 170.4, 154.7, 145.6, 139.7, 133.6, 132.3, 130.8, 130.0, 129.2, 127.6, 127.5, 126.7, 126.3, 124.1, 120.2, 70.4, 59.3, 45.4, 45.3, 42.9, 34.5, 28.2, 14.8. MS (ESI), *m/z* 365.1[M+H]$^+$.

*2-(11-(3-(dimethylamino)propylidene)-6,11-dihydrodibenzo[b,e]oxepin-2-yl)-N-propylacetamide (**33h**).*^{1}H-NMR ($CDCl_3$, 400 MHz), δ_H 7.20-7.40 (4H, m), 7.06 (1H, d, *J* = 2.2 Hz), 7.04 (1H, dd, *J* = 8.3, 2.2 Hz), 6.83 (1H, d, *J* = 8.3 Hz), 5.72 (1H, t, *J* = 7.2 Hz), 5.43 (2H, br s), 3.45 (2H, s), 3.15 (2H, q, *J* = 6.6 Hz), 2.56 (2H, m), 2.45 (2H, t, *J* = 7.2 Hz), 2.22 (6H, br s), 1.43 (2H, m), 0.83 (3H, t, *J* = 7.4 Hz). ^{13}C-NMR ($CDCl_3$, 100 MHz) δ_C 170.4, 154.7, 145.6, 139.7, 133.6, 132.3, 130.8, 130.0, 129.2, 127.6, 127.5, 126.7, 126.3, 124.1, 120.2, 70.4, 59.3, 45.3, 43.0, 41.5, 38.6, 28.1, 22.6, 11.2.MS (ESI), *m/z*379.4[M+H]$^+$.

*N-butyl-2-(11-(3-(dimethylamino)propylidene)-6,11-dihydrodibenzo[b,e]oxepin-2-yl)acetamide (**33i**).*Yield 55%.^{1}H-NMR ($CDCl_3$, 400 MHz), δ_H 7.20-7.40 (4H, m), 7.06 (1H, d, *J* = 2.2 Hz), 7.04 (1H, dd, *J* = 8.3, 2.2 Hz), 6.83 (1H, d, *J* = 8.3 Hz), 5.72 (1H, t, *J* = 7.2 Hz), 5.43 (2H, br s), 3.45 (2H, s), 3.18 (2H, q, *J* = 6.9 Hz), 2.56 (2H, m), 2.45 (2H, t, *J* = 7.2 Hz), 2.22 (6H, br s), 1.38 (2H, m), 1.24 (2H, m), 0.86 (3H, t, *J* = 7.2 Hz); ^{13}C-NMR ($CDCl_3$, 100 MHz) δ_C 170.4, 154.7, 145.6, 139.7, 133.6, 132.3, 130.8, 130.0, 129.2, 127.6, 127.5, 126.7, 126.3, 124.1, 120.2, 70.4, 59.4, 45.4, 45.4, 42.9, 39.4, 31.6, 28.2, 20.0, 13.8. MS (ESI), *m/z* 393.4[M+H]$^+$.

*2-(11-(3-(dimethylamino)propylidene)-6,11-dihydrodibenzo[b,e]oxepin-2-yl)-N-isopropylacetamide(**33j**)*. Yield 70%.^{1}H-NMR ($CDCl_3$, 400 MHz), δ_H 7.20-7.40 (4H, m), 7.06 (1H, d, *J* = 2.2 Hz), 7.04 (1H, dd, *J* = 8.3, 2.2 Hz), 6.83 (1H, d, *J* = 8.3 Hz), 5.72 (1H, t, *J* = 7.2 Hz), 5.43 (2H, br s), 3.45 (2H, s), 3.37 (2H, q, *J* = 7.1 Hz), 3.29 (2H, q, *J* = 7.1 Hz), 2.56 (2H, m), 2.45 (2H, t, *J* = 7.2 Hz), 2.22 (6H, br s), 1.11 (6H, t, *J* = 7.1 Hz); ^{13}C-NMR ($CDCl_3$, 100 MHz) δ_C 170.4, 154.7, 145.6, 139.7, 133.6, 132.3, 130.8, 130.0, 129.2, 127.6, 127.5, 126.7, 126.3, 124.1, 120.2, 70.4, 59.3, 45.3, 43.0, 41.5, 38.6, 28.1, 22.6, 22.6.MS (ESI), *m/z*379.2[M+H]$^+$.

*2-(11-(3-(dimethylamino)propylidene)-6,11-dihydrodibenzo[b,e]oxepin-2-yl)-N-(2-hydroxyethyl)acetamide (**33k**)*. Yield 67%.^{1}H-NMR ($CDCl_3$, 400 MHz), δ_H 7.20-7.40 (4H,

m), 7.06 (1H, d, J = 2.2 Hz), 7.04 (1H, dd, J = 8.3, 2.2 Hz), 6.83 (1H, d, J = 8.3 Hz), 5.72 (1H, t, J = 7.2 Hz), 5.43 (2H, br s), 3.58 (2H, m), 3.45 (2H, s), 3.30 (2H, m), 2.56 (2H, m), 2.45 (2H, t, J = 7.2 Hz), 2.22 (6H, br s); ^{13}C-NMR ($CDCl_3$, 100 MHz) δ_C 170.4, 154.7, 145.6, 139.7, 133.6, 132.3, 130.8, 130.0, 129.2, 127.6, 127.5, 126.7, 126.3, 124.1, 120.2, 70.4, 60.9, 59.6, 45.4, 45.4, 43.0, 41.4, 28.5. MS (ESI), *m/z* 381.3[M+H]$^+$.

*2-(11-(3-(dimethylamino)propylidene)-6,11-dihydrodibenzo[b,e]oxepin-2-yl)-N,N-dimethylacetamide(**33l**)*.^{1}H-NMR ($CDCl_3$, 400 MHz), δ_H 7.20-7.40 (4H, m), 7.06 (1H, d, J = 2.2 Hz), 7.04 (1H, dd, J = 8.3, 2.2 Hz), 6.83 (1H, d, J = 8.3 Hz), 5.72 (1H, t, J = 7.2 Hz), 5.43 (2H, br s), 3.45 (2H, s), 2.98 (3H, s), 2.95 (3H, s), 2.56 (2H, m), 2.45 (2H, t, J = 7.2 Hz), 2.22 (6H, br s); δ_C 170.4, 154.7, 145.6, 139.7, 133.6, 132.3, 130.8, 130.0, 129.2, 127.6, 127.5, 126.7, 126.3, 124.1, 120.2, 70.4, 59.4, 45.4, 45.4, 40.1,37.7, 35.6, 28.1.MS (ESI), *m/z* 365.2[M+H]$^+$.

*2-(11-(3-(dimethylamino)propylidene)-6,11-dihydrodibenzo[b,e]oxepin-2-yl)-N,N-diethylacetamide(**33m**)*.^{1}H-NMR ($CDCl_3$, 400 MHz), δ_H 7.20-7.40 (4H, m), 7.06 (1H, d, J = 2.2 Hz), 7.04 (1H, dd, J = 8.3, 2.2 Hz), 6.83 (1H, d, J = 8.3 Hz), 5.72 (1H, t, J = 7.2 Hz), 5.43 (2H, br s), 3.45 (2H, s), 3.37 (2H, q, J = 7.1 Hz), 3.29 (2H, q, J = 7.1 Hz), 2.56 (2H, m), 2.45 (2H, t, J = 7.2 Hz), 2.22 (6H, br s), 1.11 (6H, t, J = 7.1 Hz); ^{13}C-NMR ($CDCl_3$, 100 MHz) δ_C 170.4, 154.7, 145.6, 139.7, 133.6, 132.3, 130.8, 130.0, 129.2, 127.6, 127.5, 126.7, 126.3, 124.1, 120.2, 70.4, 59.1, 45.1, 45.1, 42.4, 40.2, 39.8, 27.7, 14.3, 13.0.MS (ESI), *m/z* 393.3[M+H]$^+$.

*N-cyclopropyl-2-(11-(3-(dimethylamino)propylidene)-6,11-dihydrodibenzo[b,e]oxepin-2-yl)acetamide (**33n**)*. Yield 66%. ^{1}H-NMR ($CDCl_3$, 400 MHz), δ_H 7.20-7.40 (4H, m), 7.06 (1H, d, J = 2.2 Hz), 7.04 (1H, dd, J = 8.3, 2.2 Hz), 6.83 (1H, d, J = 8.3 Hz), 5.71 (1H, t, J = 7.2 Hz), 5.60 (2H, br s), 3.44 (2H, s), 2.64 (1H, m), 2.56 (2H, m), 2.45 (2H, t, J = 7.2 Hz), 2.22 (6H, br s), 0.71 (2H, q, J = 7.0 Hz), 0.39 (2H, q, J = 7.0 Hz); ^{13}C-NMR ($CDCl_3$, 100 MHz) δ_C 170.4, 154.7, 145.6, 139.7, 133.6, 132.3, 130.8, 130.0, 129.2, 127.6, 127.5, 126.7, 126.3, 124.1, 120.2, 70.4, 59.4, 45.4, 45.4, 42.8, 28.2, 22.7, 6.6, 6.6.MS (ESI), *m/z* 377.2[M+H]$^+$.

2-(11-(3-(dimethylamino)propylidene)-6,11-dihydrodibenzo[b,e]oxepin-2-yl)-N-(3-

*methoxypropyl)acetamide (**33o**)*. ^{1}H-NMR ($CDCl_3$, 400 MHz), δ_H 7.20-7.40 (4H, m), 7.06 (1H, d, *J* = 2.2 Hz), 7.04 (1H, dd, *J* = 8.3, 2.2 Hz), 6.83 (1H, d, *J* = 8.3 Hz), 5.72 (1H, t, *J* = 7.2 Hz), 5.43 (2H, br s), 3.45 (2H, s), 3.30 (4H, m), 3.02 (3H, s), 2.56 (2H, m), 2.45 (2H, t, *J* = 7.2 Hz), 2.22 (6H, br s), 1.66 (2H, m). ^{13}C-NMR ($CDCl_3$, 100 MHz) δ_C 171.3, 154.7, 145.7, 139.6, 133.5, 132.4, 130.9, 130.2, 129.1, 127.5, 127.5, 126.6, 126.3, 124.2, 120.1, 71.8, 70.4, 59.4, 58.5, 45.4, 45.4, 43.0, 38.5, 28.8, 28.1.MS (ESI), *m/z* 409.3[M+H]$^+$.

*N-(4,5-dihydrothiazol-2-yl)-2-(11-(3-(dimethylamino)propylidene)-6,11-dihydrodibenzo[b,e]oxepin-2-yl)acetamide (**33p**)*.^{1}H-NMR (DMSO-d_6, 400 MHz), δ_H 12.28 (1H, s), 7.46 (1H, d, *J* = 3.6 Hz), 7.20-7.40 (4H, m), 7.19 (1H, d, *J* = 3.6 Hz), 7.06 (1H, d, *J* = 2.2 Hz), 7.04 (1H, dd, *J* = 8.3, 2.2 Hz), 6.78 (1H, d, *J* = 8.3 Hz), 5.68 (1H, t, *J* = 7.2 Hz), 5.15 (2H, br s), 3.68 (2H, s), 2.46 (2H, m), 2.35 (2H, t, *J* = 7.2 Hz), 2.07 (6H, br s); ^{13}C-NMR (DMSO-d_6, 100 MHz) δ_C 169.8, 158.5, 154.4, 145.7, 139.1, 138.1, 134.0, 132.2, 131.8, 130.5, 129.6, 128.2, 127.9, 127.3, 126.3, 123.8, 119.7, 113.9, 69.9, 59.1, 45.3, 45.3, 41.1, 27.9.MS (ESI), *m/z* 422.1[M+H]$^+$.

Table S1 Pharmacophore-based estimated activities of all synthesized compounds on PARP1 enzymatic inhibition

Compound	Enzymatic inhibition (IC50, μM)	Estimated enzymatic Inhibition (IC50, μM)
	PARP1	PARP1
**11a**	>20	54.7149
**11b**	16.17±1.24	14.8813
**11c**	4.21±2.13	15.779
**11d (PA-10)**	1.65±0.25	7.02472
**11e**	>20	76.6852
**11f**	>20	>100
**15a**	>20	>100
**15b**	>20	>100
**15c**	>20	>100
**15d**	>20	>100
**15e**	>20	>100
**19a**	13.17±2.33	16.3479
**19b**	0.75±0.27	3.38222
**19c**	>20	41.7223
**23a**	19.21±3.05	11.557
**23b**	1.04±0.17	2.14304
**23c**	>20	>100
**26a**	>20	20.6874
**26b**	17.85±2.46	43.0183
**26c**	>20	44.4732
**23a**	>20	33.5888
**23b**	>20	49.6255
**23c**	>20	48.2723
**31**	11.24±1.16	3.3436
**33a**	9.11±1.67	3.06269
**33b**	>20	>100
**33c**	>20	>100
**33d**	0.344±0.027	1.38969
**33e (OL-1)**	0.079±0.013	0.29652
**33f**	0.724±0.013	0.456771
**33g**	1.26±0.14	1.29628
**33h**	13.47±2.77	19.2644
**33i**	>20	2.14771
**33j**	7.35±1.32	9.13275
**33k**	19.12±2.16	23.8895
**33l**	4.45±1.29	3.21283

**33m**	18.91±2.29	7.89617
**33n**	>20	>100
**33o**	>20	>100
**33p**	>20	>100
**PA-10**	1.65±0.25	7.02472
**Iniparib**	n.d.	>100
**Olaparib**	0.005±0.001	0.934938

Table S2 Crystallization data and refinement statistics

Property	Value	Source
Space group	P 21 21 21	Depositor
Cell constants<br>a, b, c, α, β, γ	48.00Å 92.43Å 162.93Å<br>90.00° 90.00° 90.00°	Depositor
Resolution (Å)	50.00 - 4.01<br>37.75 - 4.01	Depositor<br>EDS
%Data completeness (in resolution range)	98.3 (50.00-4.01)<br>98.5 (37.75-4.01)	Depositor<br>EDS
R_{merge}	0.21	Depositor
R_{sym}	(Not available)	Depositor
$<I/\sigma(I)>$	7.19 (at 3.99Å)	Xtriage
Refinement program	REFMAC	Depositor
R, R_{free}	0.242 , 0.358<br>0.249 , 0.253	Depositor<br>DCC
R_{free} test set	307 reflections (5.04%)	DCC
Wilson B-factor (Å^2)	65.4	Xtriage
Anisotropy	0.934	Xtriage
Bulk solvent k_{sol}(e/Å^3), B_{sol}(Å^2)	0.29 , 11.6	EDS
Estimated twinning fraction	No twinning to report.	Xtriage
L-test for twinning	$<\|L\|>$ = 0.49, $<L^2>$ = 0.33	Xtriage
Outliers	1 of 6405 reflections (0.016%)	Xtriage
F_o,F_c correlation	0.86	EDS
Total number of atoms	5564	wwPDB-VP
Average B, all atoms (Å^2)	77.0	wwPDB-VP
